# Antimicrobial Resistance of Non-Typhoid *Salmonella* in Meat and Meat Products

**DOI:** 10.3390/foods10081731

**Published:** 2021-07-27

**Authors:** Sandra M. Rincón-Gamboa, Raúl A. Poutou-Piñales, Ana K. Carrascal-Camacho

**Affiliations:** 1 Laboratorio de Microbiología de Alimentos, Grupo de Biotecnología Ambiental e Industrial (GBAI), Departamento de Microbiología, Facultad de Ciencias, Pontificia Universidad Javeriana, Bogotá D.C. 110-23, Colombia; sandra_rincon@javeriana.edu.co (S.M.R.-G.); acarrasc@javeriana.edu.co (A.K.C.-C.); 2 Laboratorio Biotecnología Molecular, Grupo de Biotecnología Ambiental e Industrial (GBAI), Departamento de Microbiología, Facultad de Ciencias, Pontificia Universidad Javeriana, Bogotá D.C. 110-23, Colombia

**Keywords:** multidrug resistance, meat products, standard, non-typhoidal *Salmonella*

## Abstract

*Salmonella enterica* serovars are associated with numerous annual deaths worldwide and are responsible for a large number of foodborne diseases. Within this frame of reference, knowledge of antimicrobial susceptibility represents the fundamental approach of most *Salmonella* treatments. Therefore, scientific publications of antimicrobial susceptibilities and resistance must be precise, with interpretations adjusted to a particular standard. Hence, the three objectives in this study were: (i) to describe the frequency of antimicrobial-resistant isolates of Non-Typhoidal *Salmonella* (NTS) isolated from beef, pork, chicken meat, and other meat products; (ii) to describe the distribution of serovars and their multi-resistance to antibiotics for clinical use (veterinary and human) between 1996 and 2019; and (iii) to propose additional considerations that could improve the use and usefulness of the published results. Our results determined that the predominant isolates came from poultry. Enteritidis and Typhimurium were the most reported serovars by MIC (with both having the highest resistance to TET) while the lowest resistance was to CIP and CRO for Enteritidis and Typhimurium, respectively. The multi-resistance pattern AMP AMC CEP GEN KAN STR TET was the most frequently observed pattern by MIC in Montevideo and Seftenberg, while, for disc diffusion, the pattern AMP STR TET was the most frequent in the Bredeney serotype. In conclusion, researchers should carry out homogeneous sampling procedures, identify the types of the samples, use standard identification methods, and employ appropriate standards for antimicrobial susceptibility interpretation. Additionally, there is also a need for all WHO members to comply with the WHA 73.5 resolution. Our final recommendation is for all producers to reduce antibiotic prophylactic use.

## 1. Introduction

According to the World Health Organization (WHO), foodborne diseases (FBD) are diseases transmitted through contaminated food consumption. Foodborne illnesses include those caused by a microbial pathogen, parasite, chemical contaminant, or biotoxin [1]. The severity of these diseases varies from asymptomatic and mild to life-threatening, in which case life-long treatments are required. In industrialized countries, it is estimated that more than 10% of the population could suffer from a disease associated with contaminated food consumption [2]. One of the agents triggering FBD is *Salmonella* spp., which causes salmonellosis disease with a high morbidity and mortality rate in industrialized and developing countries [3].

*Salmonella* spp., is a Gram-negative bacillus with peritrichously flagella [3]. Moreover, *Salmonella* is a chemoorganotrophic organism and a facultative anaerobe, capable of using both fermentative and respiratory metabolic pathways. *Salmonella* spp. also lacks cytochrome oxidase, grows on citrate as its unique carbon source, produces sulphuric acid and decarboxylates both lysine and ornithine.

Even though it grows slowly, under favourable conditions it can survive for weeks and months in water and soils. Its optimal growth temperature is 37 °C, yet it can survive between 7 and 48 °C. Nevertheless, growth in food preserved at temperatures between 2 and 4 °C has been described. *Salmonella* spp., is viable at a pH between 4.05 and 9.5, with optimal growth at pH 6.5 and 7.5 ± 0.2 and a water activity (aw) of >0.93. These capabilities cause contamination in food-production chains, since *Salmonella* spp. can survive for a long period of time under food-stored conditions [4,5].

The *Salmonella* genus includes two species, *S*. *bongori* and *S*. *enterica* [2,3,6]. *S*. *enterica*, includes six subspecies: I, *enterica*; II, *salamae*; IIIa, *arizonae*; IIIb, *diarizonae*; IV, *houtenae*; and V, *indica* [2].

The subspecies II, IIIa, IIIb, IV and V, can be found in cold-blooded animals and the environment [4] with 1051 serovars, most designated by their antigenic formula [7].

*S. enterica* (subspecies I) strains are most commonly isolated from warm-blooded animals, including fowl and mammals, and it is the most diverse with 1586 serotypes including typhoidal and non-typhoidal species [4]. The serovar names are associated with either the disease they cause or the geographical location where they were isolated from [7], and most of them are non-pathogenic in their reservoirs [6].

Another manner of classifying *Salmonella* spp. is based on the disease it causes. Thus, it can be categorized into typhoidal and non-typhoidal varieties. The non-typhoidal *Salmonella* (NTS) are responsible for gastroenteritis [8] in various hosts. Epidemiologically, they vary in their ability to cause bacteremia and severe human diseases [9]. It has also been estimated that within the overall burden of disease, infections caused by NTS are the second leading cause of diarrheal diseases. Between 1990 and 2010, the burden calculated respectively in disability-adjusted life years (DALYs) was 180–70 per 100,000 [10].

The incubation time after ingestion of non-typhoidal *Salmonella* contaminated food is short (8–72 h). Clinical symptoms of non-typhoidal salmonellosis are associated with acute enterocolitis with abdominal pain, bloody or non-bloody diarrhea [4], nausea, and vomit. The infective dose in contaminated food or water is >10^4^ CFU. However, lower doses have been reported in high-fat foods such as meat and poultry. The disease is self-limited (from 5 to 7 days) [4,11,12]. Therefore, the worldwide non-typhoidal disease impact is high, and during the period from 1966 to 2007 there were 93.8 million cases, of which 80.3 million were associated with the consumption of contaminated food, ultimately resulting in 155,000 deaths annually [13].

Transmission of NTS to humans can occur zoonotically due to contact with feces from carrier animals or by consumption of contaminated food [3,4,11,12]. In developing countries, vegetable and water or human to human contact are the main routes of contamination. Whereas, in industrialized countries the chief source of contamination involves consumption of contaminated animal food, particularly fresh meat and eggs [4].

On the other hand, for humans, meat is a concentrated source of nutrients [13]. Therefore, new tendencies have led to the collective need for a constant supply of meat, particularly of beef and pork meat. While beef is more expensive among protein products of animal origin, in developing countries the price of pork meat is more accessible. In contrast, in developed countries, meat is a highly consumed and valued food [14]. Additionally, in the coming years, the world population could surpass 7.5 billion people, stimulating a great meat and meat product demand [15].

The contamination of meat and meat products occurs at different stages in the meat chain (processing, distribution, wholesale, manipulation, and preparation) [16]. Pathogen dissemination occurs in the abattoir, either during the evisceration process or intestinal content removal due to cross-contamination (equipment, utensils and personnel) [4,16,17,18,19].

Furthermore, the resistance of *Salmonella* spp. to one or various antimicrobial agents has drastically increased [20,21,22,23,24] as a consequence of uncontrolled use in production systems (i.e., the prevention, control, and treatment of infectious diseases) [24,25]. Moreover, its prophylactic use as growth promoters in chicken and pigs [19,20,24,26,27] has generated an even greater public health problem, resulting in therapeutic failures in disease treatment for both humans and animals [20,24].

In the European Union in 2016, resistance profiles were found in isolates for 20.4% (95, 434) of human salmonellosis. Sulfonamide/sulfamethoxazole, tetracycline, and ampicillin, and others were among the antibiotics assayed [28]. Antimicrobial resistance in NTS makes infection control and prevention more difficult. Since 1990, *S. Typhimurium* DT104 strain has increased its global dissemination. According to the National Antimicrobial Resistance Monitoring System (NARMS), in the United States of America, between 2005 and 2006, 4.1% of the isolates decreased their susceptibility to cephalosporins and 84% had resistance phenotypes to multiple antibiotics [3].

There are several methods used to test antimicrobial susceptibility such as the dilution method or minimal inhibitory concentration (MIC) and the Kirby–Bauer disc diffusion method. A combination of the former two methods is referred to as the epsilometric method (E-test) [29]. Additionally, other in vitro assays, such as agar dilution and both macro and microdilutions, have been used. The MIC and disc diffusion methods have been approved by the Clinical and Laboratory Standard Institute (CLSI) and the European Committee on Antimicrobial Susceptibility Testing (EUCAST). The standard released by both entities are frequently updated, hence the importance of following the latest editions [11].

Hence, the objectives of this work were: to describe the frequency of antimicrobial-resistant isolates of Non-Typhoidal *Salmonella* (NTS) isolated from beef, pork, chicken meat, and meat products; to describe the distribution of serovars and their multi-resistance to antibiotics for clinical use (veterinary and human) between 1996 and 2019; and to propose additional considerations that could improve the use and usefulness of published results.

## 2. Materials and Methods

### 2.1. Search Strategy

Web of Science (WoS), SCOPUS, Science Direct, and JSTOR were the websites used to provide comprehensive citation data. The interaction of three groups allowed performing search equations. The first equation included *Salmonella*/zoonotic *salmonella*/foodborne pathogen/Nontyphoid *Salmonella*; the second equation involved antimicrobial resistance, antibiotic resistance/multidrug resistance; and the third involved related meat products/ meat poultry/pork/beef by employing “AND” as the Boolean operator [30].

Regional documents were searched at Biblioteca Virtual de Salud (BVS) and PubMed. In BVS and PubMed, DECS (Descriptors in Health Sciences) and MESH (Medical Subject Headings) were utilized. The dependent terms used in the search were *Salmonella* food poisoning/ “*intoxicación alimentaria por *Salmonella* y *Salmonella* enterica*”. The independent variables associated with drug resistance were Microbial/”*facorresistencia microbiana*” and Microbial sensitivity tests/”*pruebas de sensibilidad microbiana*” [30].

To search for subjects concerning meat or meat products the terms meat products/”*productos de carne*” (in singular and plural), poultry products/”*productos avícolas*”, food safety/”*análisis de peligros y puntos críticos de control*”, “*inocuidad de los alimentos*”, food contamination/”*contaminación de alimentos*”, foodborne diseases/”*enfermedades transmitidas por los alimentos*”, fast foods/”*comidas rápidas*” and raw foods/”*alimentos crudos*” were employed [30].

### 2.2. Inclusion and Exclusion Criteria

Experimental studies performed between 1996 and 2019 were included, encompassing the year when the international observation and monitoring of antimicrobial resistance programs was initiated. English and Spanish were the selected languages, as illustrated by the search equations [30].

In the selected articles, isolates must have been from meat or meat products collected at retail or intended for the same purpose. The selected article should describe *Salmonella* spp., isolates and the non-typhoidal serotypes identification [30].

For the analysis, selected articles were classified according to the antimicrobial susceptibility detection method into the disc diffusion method and minimum inhibitory concentration (MIC). After verifying breakpoints and references, articles with interpretive criteria or isolate frequencies of resistance to antimicrobial agents were included [30].

Articles where title and abstract were not related to the topic of interest were excluded. Correspondingly, food outbreak studies were also excluded since, for many of these studies, it was difficult to identify source of contamination by *Salmonella* spp. Additionally, articles involving isolates from biological collections were not included as there was no clarity regarding sample type, years of preservation, or origin [30].

### 2.3. Extraction and Data Registry

Article assembled information included country, type of meat or meat product, the method used (disc diffusion or MIC), breakpoints, the standard used or implemented (national or international), and susceptibility test results [30].

Selected articles described the susceptibility to different families of antibiotics belonging to the following classes: penicillin, combination β-lactamase inhibitors, cephems, monobactams, aminoglycosides, quinolones, fluoroquinolones, folate pathway inhibitors, phenols, nitrofurans, and tetracyclines [30].

### 2.4. Data Analysis

Data analysis focused on three different aspects. First, in the descriptive phase, the following items were considered: sample country of origin, animal species of the meat or meat product, identified serotypes, antimicrobial susceptibility test used, and standard employed. Second, articles were classified according to the method used (disc diffusion or MIC) for each group [30].

Third, the percentage prevalence for each serotype was calculated as follows:

Calculated frequency % = Number of isolates reported/total number of isolates × 100.

The analysis was performed according to the antimicrobial susceptibility assessment method described in each study and the serovar-ties were evaluated.

Some studies used the evaluation of antimicrobial susceptibility standard for isolates of animal origin. Since 2013 the M31 version was modified into the VET01-S3 (2013). Subsequently, from June 2018, the VET 01-04 (2018) was modified into the VET08. Therefore, to maintain the article’s original information, the names of the standards were kept as published [30].

## 3. Results

### 3.1. Number of Articles, Countries, and Standard

**Figure 1:**
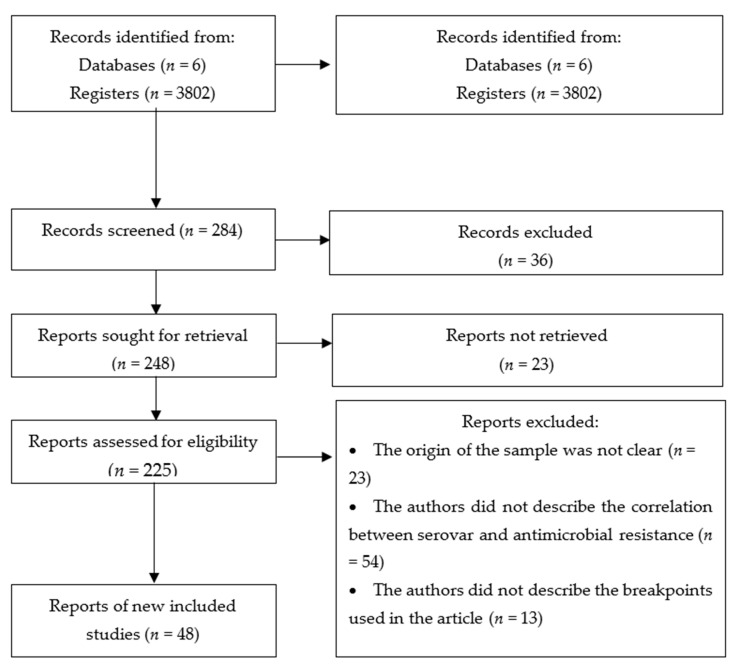
Strategy for selection of eligible articles. Flowchart made based on [31,32].

A total of 3802 articles were associated according to registered equations, of which 1141 were preselected (30%). Only 4% (48/1141) complied 100% with defined inclusion and exclusion criteria (Figure 1 and Table 1). The distribution of selected articles by country was as follows: China with seven studies (15%), followed by Egypt and Vietnam with six articles each (13%), South Korea with four papers (8%), Brazil, Canada, Colombia, Spain, United States of America, Iran, Malaysia, and Thailand with two articles each (4%) and, last, Greece, Italy, Mexico, Portugal, Rumania, Senegal, Singapore, Turkey and Venezuela with one article each (2%).

The CLSI standards were referenced in 45/48 (93.8%) articles. Thirty-seven (82.2%) of them used the M100 standard, corresponding to the Performance Standard for Antimicrobial Susceptibility Testing. Seven (15.6%) used the M31 standard for disc diffusion and dilution susceptibility Performance Standard for Antimicrobial Disc and Dilution Susceptibility Tests for Bacteria Isolated from Animals, and one (2.2%) used both M100 and M31. On the other hand, for the remaining 3/48 (6.3%) articles, interpretation criteria established by NARMS and CIPARS monitoring programs were used, as well as the international organizations “*Comité de l’antibiogramme de la société française de microbiologie*” (CA-SFM), and the European Committee on Antimicrobial Susceptibility Testing—EUCAST (Table 1).

**Table 1:** Standards used to define the interpretation criteria for antimicrobial susceptibility tests in selected articles.

	Standards Used in the Articles																						Antimicrobial Susceptibility Test	
Ref.	CLSI, M100-S9 [33]	CLSI, M100-S11 [34]	CLSI, M100-S13 [35]	CLSI, M100-S15 [36]	CLSI, M100-S16 [37]	CLSI, M100-S17 [38]	CLSI, M100-S18 [39]	CLSI, M100-S19 [40]	CLSI, M100-S20 [41]	CLSI, M100 S21 [42]	CLSI, M100-S22 [43]	CLSI, M100-S23 [44]	CLSI, M100 -S24 [45]	CLSI, M100-S25 [46]	CLSI, M100-S28 [47]	CASFM [48]	EUCAST [49]	NARMS [50]	NARMS (It Was Not Cited by Authors); CIPARS [51]	CLSI, M31-S1 [52]	CLSI, M31-A2 [53]	CLSI, M31-A3 [54]	MIC	Disc Diffusion
[55]		X																					X	
[56]																X								X
[57]				X																				X
[58]				X																	X			X
[59]				X																				X
[60]																			X				X	
[61]			X		X																			X
[62]								X															X	X
[63]					X																			X
[64]																					X		X	
[65]	X																							X
[66]																				X			X	
[67]									X														X	
[68]									X															X
[69]										X														X
[70]																						X		X
[71]																					X			X
[72]							X																	X
[73]																	X						X	
[74]										X														X
[75]					X																			X
[76]											X													X
[21]										X														X
[77]						X																		X
[78] *												X											X	X
[17]											X													X
[79]										X														X
[80]																		X				X	X	
[18]		X																						X
[81]					X																			X
[82]												X											X	X
[26]											X												X	X
[83]									X															X
[84]																						X	X	
[85]												X												X
[86]													X										X	
[87]												X												X
[19]												X												X
[88]																				X		X		X
[89]														X										X
[90]										X														X
[91]										X													X	
[92] **												X												X
[93]													X										X	
[94]											X													X
[95]													X											X
[96]												X												X
[97]															X									X

* The authors referred to the standard “CLSI, M100-S2”, and analysis for the present study used the CLSI, M100-S23. ** Authors made reference to the CLSI standard, 2013. However, it was not included in their references. Therefore, it was assumed the CLSI, M100-S23 standard was used. X means that standard or susceptibility test cited in the top of table was or were used in each study reference.

### 3.2. Reported Salmonella Enterica Serotypes

Out of the 48 analyzed studies, 211 serotypes were reported, of which 16.6% (35/211) were described five or more times (Figure 1), while the remaining 83.4% (176/211) were only reported in one to three studies (Table S1).

### 3.3. Analysis of Antibiotic Concentration Used According to Each Method

In the selected articles, the most common methodology employed was disc diffusion with 68.8% (33/48), followed by MIC 22.9% (11/48) and 8.3% (4/48), which used both methodologies (Table 1). For articles that described using MIC, in 7/15 (46.6%) of them antibiotic concentrations were not indicated [60,62,66,82,84,86,93]. Information regarding the remaining seven articles is displayed in Table S2.

### 3.4. Breakpoints and Interpretative Criteria for Antimicrobial Susceptibility Testing

Articles using MIC according to CLSI guidelines analyzed the results based on the standard issued between 2001 and 2014. Studies that implemented disc diffusion interpreted the results according to the standard published between 1999 and 2018 (Table 1).

### 3.5. Resistance to Salmonella Serotypes Assayed by the MIC Method

Six (54.5%) studies employing the MIC methodology presented results as a pattern of resistance, gathered as the sequence of antibiotics to which the microorganism was resistant, and five (45.5%) displayed the results as prevalence percentage for each antibiotic.

The number of articles that presented resistance as a percentage five, of which the work by Clemente et al., (2013) based its analysis on EUCAST (2012) clinical and epidemiological breakpoints. The highest resistance was for TET, SUL, AMP, and STR. Nhung et al., (2018) used CLSI M100-S24 clinical breakpoints, and the highest resistance percentage was for the Tetracyclines, Quinolones, Penicillins, and Folate pathway inhibitors. The resistance results for the remaining three articles are displayed in Table 2. As observed in one of them, prevalence differed from the one calculated by us (based on data) to the one published by the authors [80]. Additionally, in AMP, AMC, CIP, NAL, SXT, and TET, the calculated or reported prevalence was greater than 100 due to inconsistencies in some articles. The number of resistant isolates was higher than the number of isolates obtained as described by the authors.

In articles presenting resistance patterns, the number of total isolates was 113, classified in 18 serotypes, where Heidelberg was the predominant one. Likewise, sixty different resistance patterns were described according to the antibiotics of interest in the present work. The serotype displaying the highest number of resistances to antibiotics (10 resistances) was Bredeney [60]. The classes to which it presented the highest resistance were the aminoglycosides (GEN, TOB, AMK, KAN, STR) and cephems (CFZ, CEP, CTX, CRO, FOX, CXM, CAZ) (Table 3 and Table S3).

**Table 2:** *Salmonella* spp. antimicrobial agent frequency of resistance as determined by MIC.

Antibiotic Agent	Prevalence Analysis	Serovar (References)								
		Typhimurium * [64,66,80]	Enteritidis * [66,80]	Agona [80]	Heidelberg [66]	Indiana [80]	Infantis [66]	Kentucky [64]	Mbandaka [64]	Thompson [80]
AMP	N° isolates	148	268	46	16	114	19	24	3	55
	N° resistant isolates	97	185	22	16	105	6	5	1	66
	Reported frequency (%)			47.8	100	92.1	31.6	ND	ND	76.7
	Calculated frequency (%)	65.5	69.0	47.8	100	92.1	31.6	20.8	33.3	120
AMC	N° isolates	148	268	46	16	114	19	24	3	55
	N° resistant isolates	58	111	16	ND	91	ND	5	1	56
	Reported frequency (%)			34.8	0	79.8	ND	ND	ND	65.1
	Calculated frequency (%)	39.2	41.4	34.8	0	79.8		20.8	33.3	101.8
CRO	N° isolates	148	268	46	16	114	19	24	3	55
	N° resistant isolates	12	45	6	12	73	0	ND	ND	29
	Reported frequency (%)			15.4	75	64	ND	ND	ND	34.5
	Calculated frequency (%)	8.1	16.8	13.0	75	64				52.7
FOX	N° isolates	148	268	46	16	114	19	24	3	55
	N° resistant isolates	22	71	8	4	42	0	5	1	28
	Reported frequency (%)			17.4	25	37	ND	ND	33	32.6
	Calculated frequency (%)	14.9	26.5	17.4	25	37.0		20.8	33.0	50.9
GEN	N° isolates	148	268	46	16	114	19	24	3	55
	N° resistant isolates	40	81	6	0	93	5	ND	ND	44
	Reported frequency (%)			13.0	0	81.6	26.3	ND	ND	51.2
	Calculated frequency (%)	27.0	30.2	13.0	0	81.6	26.3			80.0
STR	N° isolates	148	268	46	16	114	19	24	3	55
	N° resistant isolates	78	155	14	16	83	18	12	1	18
	Reported frequency (%)			30.4	100	73.5	94.7	ND	33	20.9
	Calculated frequency (%)	52.7	57.8	30.4	100	72.8	94.7	50.0	33	32.7
CIP	N° isolates	148	268	46	16	114	19	24	3	55
	N° resistant isolates	22	32	10	0	93	0	ND	ND	8
	Reported frequency (%)			21.7	0	81.6	ND	0	ND	9.3
	Calculated frequency (%)	14.9	11.9	21.7	0	81.6				14.5
NAL	N° isolates	148	268	46	16	114	19	24	3	55
	N° resistant isolates	81	275	23	2	106	3	ND	ND	39
	Reported frequency (%)			50.0	12.5	93	15.8	ND	ND	45.4
	Calculated frequency (%)	54.7	102.6	50.0	12.5	93.0	15.8			70.9
SXT	N° isolates	148	268	46	16	114	19	24	3	55
	N° resistant isolates	70	123	23	0	105	2	ND	ND	60
	Reported frequency (%)			50.0	0	92.9	10.5	0	ND	69.8
	Calculated frequency (%)	47.3	45.9	50.0	0	92.1	10.5			109.1
TET	N° isolates	148	268	46	16	114	19	24	3	55
	N° resistant isolates	123	191	30	0	104	0	15	0	70
	Reported frequency (%)			65.2	0	91.2	ND	ND	ND	81.4
	Calculated frequency (%)	83.1	71.3	65.2	0	91.2		62.5		127.3

No. isolates, number of isolates; No. resistant, number of resistant isolates; F. Reported (%), frequency reported; F. Calculated (%), frequency calculated. The box highlighted in yellow are the calculated values that exceeded 100%, while the boxes highlighted in gray indicate the values that could not be calculated. * For Enteritidis and Typhimurium serovars, frequency was calculated based on the sum of reported isolates in the articles.

### 3.6. Salmonella Serotype Resistance Assayed by the Disc Diffusion Method

Resistance and multi-resistance analysis were reported on 31/33 (93.9%) of the studies. For 2/33 (6.1%), isolate, pattern or resistance percentage, and isolate origin were not related [63,68]. On the other hand, of the 31 articles analyzed, 6 (19.4%) presented the results as resistance percentage, 19 (61.3%) as the pattern of resistance, and 6 (19.4%) as the pattern and resistance percentage.

The articles that presented resistance results as the percentage of prevalence were 12/31 (39%), of which Valdezate et al. (2007) showed resistance percentage to the Enteritidis and Typhimurium; serotypes, describing the highest resistance to spectinomycin with 99% (95/96) and 87% (46/53), respectively, followed by NAL 40.6% (39/96) for Enteritidis and TET with 71.7% (38/53) for Typhimurium. The remaining 11 studies are displayed Table 4 and Table S4, where resistance percentage calculated for reported serotypes in more than one study appear (the ones with only one serotype are in Table S5). It is noteworthy that in various studies [17,83,92], some of the reported prevalences differ from those calculated by us. Additionally, the serotype most frequently found was Typhimurium (8/11 (72.7%)), and the most frequent resistance for this serotype was tetracycline.

Multi-resistance pattern analysis allowed the identification of 692 isolates, of which 444 presented different patterns (Table S6). The remaining 248 isolates were grouped in 27 different patterns (Table 5). The profile AMP SXT STR TET was the most prevalent resistance pattern in these isolates. Additionally, Enteritidis and Typhimurium serotypes displayed a resistance pattern with the greatest number of antibiotic classes: penicillins (AMP), β- lactamase inhibitors (AMC), cephems (CTX CRO FOX CPD), monobactams (ATM), aminoglycosides (GEN KAN STR), fluoroquinolones (CIP), quinolones (NAL), folate pathway inhibitors (SXT), phenolic compounds (CHL), and nitrofurans (TET).

**Table 4:** Frequency of *Salmonella* isolates resistant to antimicrobials as determined by disc diffusion.

Serovar	Number of Isolates	Calculated Frequency of Resistant Serovar (%)													Ref.
		AMP	GEN	AMK	KAN	STR	CIP	OFX	NAL	SXT	TMP	SUL	CHL	TET	
Agona	71	22.5								28.2	28.2		23.9	39.4	[79]
	1	100							100	100				100	[87]
	56	16.1		1.8		16.1	1.8	91.1	19.6	17.9		41.1	89.3	48.2	[92]
Anatum	5	100	20.0		20.0	60.0	40.0		40.0				60.0		[17]
	7	100							57.1	85.7			57.1	100	[87]
	3				33.3				33.3	66.7			66.7	100	[97]
Corvallis	1	100													[56]
	46	17.4				17.4	17.4	87.0	43.5	17.4		73.9	52.2	80.4	[92]
	3					100								100	[97]
Derby	11	9.1				45.5				18.2		81.8	18.2	100	[59]
	1					100								100	[83]
	1	100											100	100	[87]
	75	56.0	36.0	1.3		36.0	33.3	42.7	40.0	42.7		92.0	44.0	81.3	[92]
	79	41.8	24.1	2.5	25.3	45.6	25.3		40.5	43.0			35.4	77.2	[97]
Enteritidis	6	33.3				33.3			66.7	33.3	16.7			66.7	[56]
	6					16.7			66.7						[61]
	27	55.6	44.4		33.3	59.3			92.6	3.7			7.4	44.4	[69]
	30	86.7	26.7		46.7	86.7	73.3		73.3				80.0		[17]
	25	8.0			44.0	56.0			88.0	52.0	68.0			48.0	[81]
	7	100	14.3	14.3	14.3	71.4	14.3		100	28.6			14.3	28.6	[97]
Hadar	6	16.7				100				50.0	50.0	50.0		100	[56]
	8	25.0				37.5			87.5		62.5		12.5	87.5	[61]
	31	25.8			3.2					22.6	22.6		9.7	74.2	[79]
	6	16.7			66.7	50.0			100	83.3	66.7		16.7	100	[81]
Indiana	28	71.4	46.4		14.3		39.3	39.3		57.1	57.1		78.6	85.7	[79]
	5								100				20.0	100	[87]
	4	100	50.0	75.0	100	50.0	100		100	75.0			100	100	[97]
Kentucky	27	44.4				18.5	3.7		11.1	33.3	29.6	25.9		22.2	[56]
	2	100							100				100		[87]
	38	21.1	7.9	2.6		13.2	28.9	65.8	28.9	68.4		92.1	78.9	86.8	[92]
London	57	61.4	57.9			68.4		15.8	1.8	70.2		77.2	42.1	86.0	[92]
	20	50.0	25.0		5.0	45.0				50.0			35.0	50.0	[97]
Mbandaka	3										33.3	100			[56]
	34		2.9	2.9		73.5	2.9	61.8	2.9	5.9		100		97.1	[92]
Muenster	12	50.0				33.3				75.0	75.0	75.0		33.3	[56]
	10	90.0	20.0		20.0	70.0	40.0		60.0				60.0		[17]
Panama	12	83.3	8.3			75.0			50.0	33.3		83.3	75.0	75.0	[59]
	8	100							12.5	100			100	100	[87]
Rissen	6	100							33.3	50.0			33.3	100	[87]
	81	77.8	2.5			19.8	1.2	8.6	7.4	77.8		77.8	8.6	95.1	[92]
	18	55.6	5.6		5.6	22.2	5.6		5.6	77.8			5.6	77.8	[97]
Schwarzengrund	2	100								100	100	100		100	[56]
	7	100							100	14.3			100	14.3	[87]
Stanley	6	83.3							50.0					83.3	[87]
	3					33.3	33.3						33.3	33.3	[97]
Thompson	83	2.4				54.2			96.4		77.1			81.9	[61]
	54	13.0			31.5	59.3			96.3	72.2	75.9		3.7	90.7	[81]
	3	33.3				33.3	33.3		33.3	33.3			33.3	33.3	[97]
Typhimurium	8	62.5	12.5			12.5			50.0	12.5		37.5	37.5	87.5	[59]
	3					33.3			66.7		66.7			100	[61]
	3					100									[69]
	40	100	27.5		45.0	95.0	67.5		82.5				92.5		[17]
	14	14.3			35.7	57.1			85.7	64.3	78.6			85.7	[81]
	5	40.0	100			80.0	40.0		80.0	100			60.0	100	[83]
	84	83.3	15.5	1.2		59.5	13.1	29.8	29.8	27.4		89.3	40.5	82.1	[92]
	22	72.7	9.1	4.5	36.4	36.4	4.5		81.8	50.0			50.0	63.6	[97]

### 3.7. Serotype Resistance Determined by MIC and Disc Diffusion Methods

The total number of articles using two methods for antimicrobial susceptibility evaluation was 4/48 (8.3%), of which three presented results as resistance pattern and one presented the results as a percentage of prevalence.

For one article [78], the highest resistance (94%) was observed for the Heidelberg serotype to TET. Additionally, when comparing the reported percentage in the article and the calculations according to the data published by the authors, an inconsistency was observed for AMC, for the serotypes Enteritidis (Reported: 37.31 and Calculated: 7.5), and for Typhimurium (Reported: 2.50 and Calculated: 5).

Observed multi-resistance patterns are displayed in Table 6. The serotype with the highest resistance was Heidelberg, which was resistant to six classes of antibiotics and nine antibiotics (AMP AMC CFZ CTX CRO FOX CIP NAL TET), where TET was the antibiotic presenting resistance more frequently in isolates.

## 4. Discussion

### 4.1. Number of Articles, Countries, and Standard

A total of 48 articles were included in this study after eliminating those based on exclusion criteria (Figure 1), where the most representative countries were China, Egypt, and Vietnam. On the other hand, the most implemented standard was the CLSI M100 (Table 1). However, the Clemente et al., (2013) study used ECOFFs clinical and epidemiological cut-off points for bacterial resistance analysis.

### 4.2. Salmonella Enterica Reported Serotypes

Among the 48 studies selected, 211 serotypes were identified. This diversity was due to regional differences, environmental factors, origin, and food production practices. These factors favour the specific serovars found by influencing survival or transmission routes [98].

**Figure 2:**
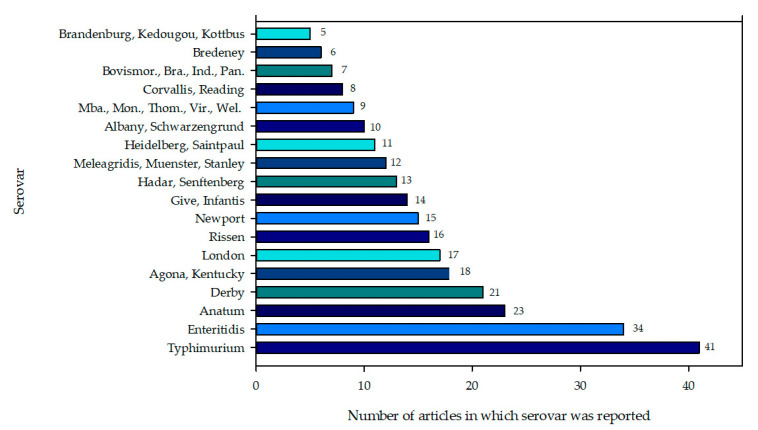
Frequency of serovars described in more than five studies. Abbreviations: Bovismor, Bovismorbificans; Bra, Braenderup; Ind, Indiana; Pan, Panama; Mba, Mbandaka; Mon, Montevideo; Thom, Thompson; Vir, Virchow; Wel, Weltevreden.

The most frequently recognized serovars were four (Figure 2). Between 2004 and 2016, Typhimurium caused many of the clinical infections worldwide [98]. It is also one of the most common causative agents for invasive salmonellosis by non-typhoid serotypes [99]. This serotype also affects livestock production systems and has been isolated in poultry, pigs, and cattle, this latter being the principal carrier [100].

The second most frequent serotype was Enteritidis (Figure 2), the principal agent causing clinical salmonellosis reported between 2004 and 2016 in Brazil, Canada, Europe, China, the United States of America, Tunisia, and Thailand [98]. *S. enteritidis* transmission to humans occurs through poorly cooked chicken, contaminated raw eggs, or products prepared with contaminated eggs. In poultry, this serovar can remain in the reproductive system tissue. Hence, it the necessary to control this microorganism during poultry production [101,102].

*S.* Anatum was the third serotype reported (Figure 2). This serotype was described first in 2005 as the ninth serotype resulting in human illnesses on the European Food Safety Authority report of tendencies and zoonosis, zoonotic agents, and outbreaks associated with food. *S.* Anatum is one of the serotypes responsible between 2015 and 2017 for an increase in human infections in Taiwan. Many of its isolates are multiresistant and prevalent in animal species destined for human consumption [103].

The fourth serotype reported and the most frequently found in selected articles was *S.* Derby (Figure 2). This serotype has been described in pork production, where pork meat is the route of transmission in some human outbreaks [104]. It has also been frequently reported in pig slaughter, surviving in first minute during the scalding process [105].

The remaining 64 serotypes appear in Figure 2, Table S1; 16/64 (25%) were found in at least ten or more selected articles. These 16 serotypes had been previously notified by NARMS (1997–2015) [106,107,108,109,110,111,112,113,114,115,116,117,118,119,120,121,122,123,124,125], EFSA (2004–2015) [106,107,108,109,110,111,112,113,114,115,116,117,118,119,120,121,122,123,124,125] and EFSA (2004–2015) [126,127,128,129,130,131,132,133,134] reports as serotypes of importance in human salmonellosis (Agona, Kentucky, Newport, Hadar, Stanley, Heidelberg, and Saint Paul). Therefore, in order to monitor these serotypes, surveillance of meat and meat products (source of propagation) is required.

### 4.3. Antibiotic Concentration Analysis According to Each Method Used

The disc diffusion method used in 68.8% of the evaluated studies represents a standard test in various diagnostic laboratories. It is easy to set up, and has a low cost [135,136]. However, to determine MIC in resistance monitoring programs, quantitative tests are preferred due to their accuracy [135,136]. Nonetheless, in the selected reports, MIC analysis was described only for 22.9% of them.

### 4.4. Salmonella Serotype Resistance Evaluated Using MIC

Among the most used analysis strategies described in the WHO’s “Integrated surveillance of antimicrobial resistance in foodborne bacteria” [137] guide is the percentage calculation of resistant, intermediate, and susceptible isolates. These, in turn, can be stratified as a function of isolation date, geographical location, origin or the frequency of isolations in population studies, allowing for the detection of pathogen’s behaviour in different environments.

In this work, when comparing the MIC methodology, 45.5% of the studies (5/11) reported results as a percentage of prevalence. Of these, in the Clemente et al., (2013) study, 76/127 (59.8%) of the isolates presented serotype I4 [5],12:i:-, which were resistant to AMP STR SUL TET in compliance with the ASSuT phenotype. Within this serotype, 21.3% presented co-resistance to other antibiotics, such as GEN and CHL, demonstrating the presence of multiresistant isolates. Furthermore, three isolates obtained from pork meat products had the blaCTX-M gene, indicating that β-lactamase spread through the food production chain.

In the study by Nhung et al., (2018) isolates from chicken and beef meat were identified; 11 out of 12 isolates with the Kentucky ST 198 serotype were multiresistant. They found resistance to diverse classes of antibiotics such as Cephems, Monobactams, Penicillins, Quinolones, Tetracyclines, and Phenolic compounds. *S.* Kentucky ST 198 is resistant to cephalosporins, carbapenems, and heavy metals mediated by plasmids. In contrast, resistance to quinolones is due to mutations in topoisomerase encoding genes (*gyr*A, *gyr*B, *par*C, *par*E). For both cases, resistance resulted from indiscriminate antibiotic prophylactic use in chickens, generating selective pressure [138].

The other three studies are shown in Table S1, where the serotypes Agona, Heidelberg, Indiana, Infantis, Kentucky, Mbandaka, and Thompson were described only once. Serotypes Enteritidis and Typhimurium were reported in two and three studies, respectively. Isolates belonging to S. Enteritidis showed the most resistance to NAL, followed by TET, AMP, and STR. In *S. Typhimurium* isolates, the most resistant profile was for TET, followed by AMP, NAL, and STR. *Salmonella* serotypes resistant to Tetracycline have been frequently described in the scientific literature due to the prophylactic use in livestock production, where the most common genes identified are *tet*A and *tet*B [139,140,141]. Nonetheless, in certain countries, Tetracycline use in livestock production is prohibited.

On the other hand, for the 54.5% (6/11) of the studies using MIC (Table 3 and Table S3), the serotype with the highest number of resistance (six classes and ten antibiotics) was the Bredeney serotype reported by Cook et al., (2009). The same study described that this serotype, as previously reported by CIPARS 2003, is frequently observed in clinical isolates from turkey meat. Moreover, the variety of resistances detected by Cook et al. it is noteworthy (2009).

It is also necessary to point out, in the studies performed by Cook et al., (2009) and Gad et al., (2018), a multi-resistance profile was observed which was similar to the Enteritidis, Anatum_var-15+, Montevideo, Saintpaul, and Seftenberg serotypes for the Penicillins, β-lactams, Cephems and Aminoglycosides antibiotics classes (Table 3 and Table S3), where resistance to β-lactams and Aminoglycosides were similar to the ones reported in cattle by Gad et al., (2018), suggesting that containment measures did not work.

*S*. *Typhimurium* and Enteritidis isolates described in the study by Ahmed et al., (2014) demonstrated a more complex resistance pattern (Table 3 and Table S3), with resistance to 15 antibiotics from 10 different classes in chicken and beef products.

The study also identified antimicrobial resistance molecular mechanisms, among which the integron Class 1 genes (*aadA2* and *bla*_PSE-1_ genes), genes coding for β-lactamases (*bla*_TEM-1_, *bla*_CMY-2_, *bla*_CTX-M-3_), genes with resistance to Quinolones (*qnrB*, *aac(6′)-Ib-cr*), and a gene with resistance to Florfenicol (*flo*R) were observed. These findings demonstrate how challenging a treatment would be for a patient infected with such an isolate.

### 4.5. Salmonella Serotype Resistance Evaluated Using Disc Diffusion

Table 4 and Table S4 shows that resistance to AMP and CHL was present in all studies, possibly due to the frequent antibiotic use as a prophylactic. These are low-cost, accessible and commonly used antibiotics in some countries for human and poultry production without “medical prescriptions”. Additionally, in the poultry industry, these medications are used as growth promoters and for therapeutic purposes, which trigger antimicrobial resistance in enteric bacterial microbiota. Subsequently, it can be transferred (horizontally and or vertically) to *Salmonella* spp. isolates, allowing multiresistant strains to survive and spread out and ultimately resulting in a public health problem [56,83].

Resistance to Quinolones (NAL) occurred in 10/11 studies (90.9%) in the Typhimurium (6 articles), Enteritidis (5), Kentucky (3), Panama (2), Hadar (2), Anatum (2), Thompson (2), Derby (2), Rissen (2), Muenster (1), Agona (1), Albany (1), Indiana (1), Stanley (1), Schwarzengrund (1), London (1), Mbandaka (1), Corvallis (1) serotypes. Resistance to NAL in Enteritidis and Typhimurium serotypes is considered a public health problem due to human antibiotic use [59]. Furthermore, the use of NAL in human enteric infections has been reconsidered in countries such as Iran [61].

Some countries such as South Korea and Iran use Cephems, Quinolones, and Aminoglycosides (growth promoters or treating bacterial infections) in the poultry industry [69,81,142]. Moreover, the perception regarding Quinolones resistance has changed. For example, in the study by Bada-Alambedji et al., (2006), Quinolones resistance was considered an alarm signal and the last therapeutic resource against *Salmonella* multiresistant isolates. In contrast, in the study by Yang et al., (2019) it was described as one of the most frequently observed antimicrobial resistances.

Resistance to TET was observed in 10/11 studies (90.9%). This antibiotic has been frequently used as a prophylactic growth promoter and food supplement in poultry productions in Iran and porcine productions in Brazil [59,61,92,97]. For humans, the Tetracycline antibiotic class represent a frequent selection in the clinic, hence the importance of surveillance of resistant isolates in livestock production and finished product [143].

Furthermore, as illustrated in Table 5, 248 isolates that were multi-resistant were grouped in 27 different patterns. Noteworthy were AMP and TET, which were present in most patterns. In contrast, NAL, STR, and SXT were uncommon.

NAL resistance is associated with poultry production, due to Quinolone use as a prophylactic [70]. Nevertheless, NAL has also been observed in beef and pork meat [58,72,74,87,95]. STR is not a therapeutic agent against *Salmonella* infections. However, resistance to this antibiotic is an epidemiological marker of the ACSSuT pattern, associated with *S. enterica* serovar Typhimurium phage type DT104 [144].

The AMP SXT TET pattern suggests that these isolates have three different resistance mechanisms (inhibition of cell wall synthesis, inhibition of metabolic factors, and inhibition of protein synthesis, respectively). Therefore, treatment could be unsuccessful resulting in a public health problem, especially when the isolates come from food products [77].

In addition, the emergent CHL resistance in Brazil [59] and Europe (Council Regulation (EEC) No. 2377/90, Annex IV) [145] resulted in its ban from use in livestock. Hence, the council established zero tolerance to CHL and nitrofurans in food from animal origin, because residues are toxic (causing carcinogenicity and mutagenicity). It has also been reported that antibiotic use alters the resistome of exposed microbial communities and that this effect can persist for decades even after use has ceased [71].

Herein, AMP CHL SXT TET was one of the most common antibiotic patterns and was part of other more complex patterns present in more than one study. Penicillin, sulphonamides, and tetracyclines are the first antimicrobial choice against bacterial infection in poultry diseases. However, their use as a prophylactic can pressure the persistence of resistant phenotypes [95]. Nevertheless, in various studies [74,85,87,89], isolates were also obtained from pork and beef meat.

Finally, in the studies conducted by Gharieb et al. (2015) and Sodagari et al. (2015), the resistance pattern to antibiotics could be different if the authors employed the standard described for the optimal period for CRO, CTX, IMI, and CIP antibiotics.

### 4.6. Salmonella Serotype Resistance Determined by MIC and Disc Diffusion

Thirteen multi-resistance patterns were determined by MIC and disc diffusion techniques (Table 6), as reported by Molina et al., (2010), Donado-Godoy et al., (2015) and Choi et al., (2015). In the study by Molina et al., (2010), the disc diffusion assay was used to evidence β-lactamase, employing the synergism of double-disc (AMC CAZ CRO CTX ATM). Hence, the thirteen-antimicrobial resistance patters (Table 6) came from two different techniques.

The most frequently observed antibiotics (Table 6) were AMP, CTX, and TET, followed by CFZ and NAL. The presence of CTX in ten resistance patterns are noteworthy because this is a third-generation cephalosporin (of extended-spectrum and recent synthesis). Additionally, in the article by Donado-Godoy et al., (2015), two isolates of the Heidelberg serotype presented resistance to CTX and CRO (Table 6). Heidelberg serovar is emerging as pathogenic in humans, either due to FBD or zoonosis, and is more invasive than other serovars causing gastroenteritis. Additionally, it generates complications such as septicemia, myocarditis, extra-intestinal infections, and death. A significant temporal correlation has also been demonstrated between the prevalence of Ceftiofur-resistant S. Heidelberg in chicken and human meat in Quebec (Canada) [146,147].

Resistant *Salmonella* to third and fourth-generation Cephalosporins became a health problem since Cephalosporins are the choice-treatment against invasive salmonellosis in immunosuppressed patients when salmonellosis is due to Fluoroquinolone resistant *Salmonella*. Likewise, CRO use has intensified in the treatment against infections in children due to CRO’s pharmacokinetic properties [75,79,82].

As with other antibiotics, the increase in *Salmonella* isolates resistant to Cephalosporins of a broad-spectrum could be conditioned to the use of veterinary treatments. Such is the case for feeding cattle, where Ceftiofur is used in respiratory diseases. It is also used in production practices implemented in some countries such as Japan, where Ceftiofur was used as a disinfectant in chicken embryos and hatched chickens until March 2012 [62,148,149]. Even though this is the case, Ceftiofur use has decreased or has been eliminated in animal production. The problem, however, has worsened since some serovars, such as the Manhattan, maintain the IncX1 plasmid carrying the *blaTEM-52* gene [148].

Finally, food production of animal origin is currently changing due to pressure for safer food and new guidelines on the marketing of food of animal origin, among other reasons. Consequently, in some countries, there are already restrictions on the use of antibiotics in animal production. In some cases, producer associations have voluntarily eliminated antibiotics in animal production. In Canada, the chicken industry has voluntarily removed the preventive use of some antimicrobials including Ceftiofur. In the United States, some producers have opted not to use antibiotics in production, initiatives referred to as either “no antibiotics ever” (NAE) or “raised without antibiotics” (RWA) [142,150,151].

## 5. Conclusions

The isolates on the articles herein selected came from poultry, cattle, and pigs, where poultry was the predominant source (60% articles). Enteritidis and Typhimurium were the most reported serovars, as evidenced by MIC antimicrobial resistance.

For S. Enteritidis, the highest percentage of resistance was against TET and the lowest was to CIP, while for Typhimurium the highest percentage of resistance was against TET and the lowest was to CRO. The most frequent MIC multi-resistance pattern was against AMP AMC CEP GEN KAN STR TET (five antimicrobial classes and seven antibiotics), observed in Montevideo and Seftenberg. For disc diffusion, the most frequent multi-resistance pattern was AMP STR TET, detected in five articles, specifically for the Bredeney serovar (20%). In the only study that used both MIC and disc diffusion, authors only described percentage of resistance. No similar pattern was observed in the other studies.

Researchers should have more homogeneity in presenting results, sampling procedures, sample type, identification methods, and selection of the appropriate standard [30], as the aforementioned affect the frequency of detection for *Salmonella* spp., [58].

Lastly, it is necessary to punctuate the importance of Resolution WHA 73.5 compliance for all countries which are members of the WHO [152]. Furthermore, producers of the different guilds must reduce antibiotic prophylactic use in their production.

## Figures and Tables

**Table 3:** Patterns of multidrug resistant *Salmonella* spp. as determined by MIC.

Ref.	Pattern	Serovar/ (Total Serovar)	Total Isolates with the Pattern	% Isolates with the Pattern
[67]	AMP SXTTET	Rissen (1)	1	100
		Worthington (1)	1	100
[91]	AMC CEP GEN KAN STR	Ouakam (1)	1	100
		Tennessee (7)	2	29
[60]	AMP AMC CEP FOX	Agona (3)	3	100
		Heidelberg (13)	2	15
		Litchfield (2)	2	100
[91]	AMP AMC CEP GEN KAN STR	Anatum (1)	1	100
		Tennessee (7)	4	57
[91]	AMP AMC CEP GEN KAN STR NAL TET	Enteritidis (3)	1	33
		Saintpaul (3)	1	33
[91]	AMP AMC CEP GEN KAN STR TET	Montevideo (1)	1	100
		Anatum_var-15+ (1)	1	100
		Saintpaul (3)	2	67
		Seftenberg (4)	2	50
[67]	AMP AMC CRO FOX	Heidelberg (13)	11	85
		I:ROUGH-O:r:1,2 (1)	1	100
		Infantis (4)	1	25
		Kentucky (2)	2	100
[21]	AMP AMC CTX CRO FOX CPD ATM GEN KAN STR CIP NAL SXT CHL TET	Typhimurium (4)	1	25
		Enteritidis (3)	1	33
[91]	AMP AMC GEN KAN STR	Seftenberg (4)	1	25
		Tennessee (7)	1	14
[60,91]	AMP CEP GEN	Enteritidis (3)	1	33
		Seftenberg (4)	1	25
[21]	AMP KAN NAL SXT CHL TET	Typhimurium (4)	2	50
		Typhimurium (4)	1	25
[86]	GEN TOB AMK CIP SXT	Enteritidis	ND	0
		Kentucky	ND	0
[84]	STR CIP NAL SUL TET	Infantis (4)	3	75

**Table 5:** Patterns of multidrug resistant *Salmonella* serovar as determined by disc diffusion.

Ref.	Pattern	Serovar/ (Total Serovar)	Total Isolates with the Pattern	% of Isolates with the Pattern
[71]	AMC CEP NAL	Typhimurium (34)	1	3
[69]		Montevideo (3)	1	33
[70]	AMP CEP NAL TET	Hadar (1)	1	100
[56]		Tshiongwe (1)	1	100
[70]	AMP GEN KAN STR CIP SXT TET	Enteritidis (25)	2	8
[21]		Infantis (4)	1	25
[74]	AMP GEN NAL SXT TET	Anatum (20)	1	5
[70]		Typhimurium (34)	1	3
[74]	AMP GEN STR SXT CHL TET	Derby (70)	1	1
[85]		London (3)	1	33
[72]		Panama (9)	1	11
[89]		Typhimurium (34)	1	3
[74]	AMP KAN STR NAL SXT CHL TET	Meleagridis (9)	1	11
[21]		Typhimurium (34)	1	3
[56]	AMP NAL CHL	Kentucky (7)	2	29
[87]		Schwarzengrund (7)	6	86
[87]	AMP NAL CHL TET	Albany (5)	1	20
		Anatum (20)	1	5
[18]		Derby (70)	1	1
[76]	AMP NAL SXT	Enteritidis (25)	1	4
[18]		Kentucky (7)	4	57
		Muenster (6)	6	100
		Virchow (2)	2	100
[87]	AMP NAL SXT CHL	Schwarzengrund (7)	1	14
[74]		Anatum (20)	1	5
		Infantis (4)	1	25
[74]	AMP NAL SXT CHL TET	Derby (70)	1	1
		Infantis (4)	1	25
[87]		Albany (5)	4	80
		Anatum (20)	2	10
		Panama (9)	1	11
		Rissen (4)	1	17
[95]		Brancaster (3)	1	33
		Stanley (8)	1	13
[87]	AMP NAL SXT TET	Agona (3)	1	33
		Anatum (20)	1	5
[74]		Derby (70)	1	1
[70]		Typhimurium (34)	1	3
[74]	AMP NAL TET	Derby (70)	4	6
[87]		Rissen (4)	1	17
		S.4.5.12:I: −(2)	2	100
		Stanley (8)	3	38
[81]		Enteritidis (25)	1	4
		Typhimurium (34)	2	6
[58]		Anatum	ND	ND
[70]		Typhimurium (34)	2	6
[72]	AMP STR NAL SXT TET	Anatum (20)	1	5
		Derby (70)	2	3
[85]		Derby (70)	2	3
[85]	AMP STR SXT CHL TET	Meleagridis (9)	2	22
[74]		Typhimurium (34)	1	3
		Derby (70)	1	1
[89]		Rissen (4)	1	17
		Give (1)	1	100
		Typhimurium (34)	1	3
[72]	AMP STR SXT TET	Agona (3)	1	33
		Brandenburg (1)	1	100
		Saintpaul (2)	1	50
[74]		Derby (70)	1	1
[72]	AMP STR TET	Derby (70)	7	10
		Typhimurium (34)	4	12
[85]		Derby (70)	7	10
		Typhimurium (34)	4	12
[74]		Derby (70)	7	10
		Meleagridis (9)	1	11
		Newport (5)	1	20
[89]		Derby (70)	7	10
		Typhimurium (34)	4	12
[70]		Bredeney (3)	1	33
[74]	AMP SXT CHL TET	Typhimurium (34)	3	9
[87]		Anatum (20)	1	5
		Panama (9)	7	78
[81]		Bovismorbificans (1)	1	100
[95]		Brancaster (3)	1	33
		Stanley (8)	4	50
		Typhimurium (34)	3	9
[77]	AMP SXT TET	Agona (3)	1	33
		Anatum (20)	5	25
		Bredeney (3)	2	67
		Coeln (1)	1	100
		Derby (70)	8	11
		London (3)	1	33
		Senftenberg (1)	1	100
		Typhimurium (34)	1	3
[85]		Derby (70)	8	11
[74]		Derby (70)	8	11
[87]		Anatum (20)	5	25
		Rissen (4)	1	17
[71]	CEP STR NAL	Enteritidis (25)	4	16
		Newport (5)	1	20
[69]		Enteritidis (25)	4	16
		Montevideo (3)	2	67
[70]	KAN STR NAL TET	Blockey (4)	4	100
[81]		Enteritidis (25)	3	12
		Newport (5)	2	40
[85]	NAL CHL TET	Derby (70)	1	1
		Typhimurium (34)	4	12
[87]		Indiana (1)	1	100
[74]	NAL SXT TET	Infantis (4)	1	25
		Meleagridis (9)	5	56
[81]		Thompson (1)	1	100
[71]	STR NAL TET	Enteritidis (25)	5	20
[72]		Anatum (20)	1	5
		Derby (70)	1	1
		Reading (1)	1	100
[81]		Enteritidis (25)	5	20
		Newport (5)	1	20
[58]		Hadar	ND	ND
[56]	STR SUL TET	Brancaster (3)	1	33
[58]		Anatum	ND	ND
		London	ND	ND
[72]	STR SXT CHL TET	Anatum (20)	1	5
		Derby (70)	1	1
		Saintpaul (2)	1	50
[74]		London (3)	1	33
[75]	STR SXT SUL TET	Derby (70)	1	1
[56]		Kentucky (7)	1	14

**Table 6:** *Salmonella* serovar patterns of multidrug resistance as determined by disc diffusion and MIC.

Reference	Multiresistance Patterns	Serovar (Total Isolates)	Total Isolateswith the Multiresistance Pattern	% Frequency of Multiresistance Pattern
[62]	AMP CTX SXT	Enteritidis (3)	1	33.3
[26]	CIP NAL TET	Heidelberg (11)	2	25.0
	STR SXT TET	Typhimurium (1)	1	100
	STR CIP NAL TET	Heidelberg (11)	1	9.1
[82]	AMP AMC CFZ CEP CTX TET	Bareilly (1)	1	100
[62]	AMP CFZ CTX ATM CIP SXT	Heidelberg (11)	2	18.2
	AMP TZP CFZ CTX ATM SXT	Heidelberg (11)	1	9.1
[82]	AMP CFZ CEP CTX STR NAL TET	Infantis (8)	8	100
		Virchow (16)	8	50
[82]	AMP CFZ CEP CTX GEN STR NAL TET	Enteritidis (3)	1	33.3
		Richmond (3)	3	100
[26]	AMP AMC CFZ CTX CRO FOX NAL TET	Heidelberg (11)	3	27.3
[82]	AMP AMC CFZ CEP CTX STR NAL TET	Virchow (16)	7	43.8
[82]	AMP AMC CFZ CEP CTX GEN STR NAL TET	Enteritidis (3)	1	33.3
		Virchow (16)	1	6.3
[26]	AMP AMC CFZ CTX CRO FOX CIP NAL TET	Heidelberg (11)	2	18.2

## Data Availability

Data are contained within the article.

## Supplementary Materials

The following are available online at https://www.mdpi.com/article/10.3390/foods10081731/s1, Table S1. Serovars that were reported in 1 to 3 studies. Table S2. The concentration of antimicrobial agent used in the antimicrobial susceptibility test by MIC. Table S3. Multi-resistance patterns reported once using MIC technique. Table S4. Calculated antimicrobial susceptibility frequency rates of *Salmonella* serovars using the disc diffusion technique. Table S5. Reported resistance percentages using disc diffusion method. Table S6. Multiresistance patterns described only one for each serovar using the disk diffusion technique.

## Abbreviations

Abbreviations for antimicrobial agent reported in the different selected articles and standardized abbreviation.

**Antimicrobial Agent**	**Abbreviations in the Reports**	**Abbreviations in This Work**
Penicillins		
Ampicillin	AM, AMP, A, Amp, Ap	AMP
β-Lactam/β-Lactamase inhibitor combinations		
Amoxicillin/clavulanate	AUG, AMC, Amc, AC	AMC
Ampicillin-sulbactam	SAM, AS	SAM
Piperacillin-tazobactam	PPC-TAZ, TZP	TZP
Cephems		
Cefazolin	CFZ, KZ, CZ, CF, CZD	CFZ
Cephalothin	CF, CEP, CEF, KF	CEP
Cefepime	CPM, FEP	FEP
Cefotaxime	CTX, TAX, CT	CTX
Ceftriaxone	AXO, CRO, Co, CTR	CRO
Cefoxitin	FOX	FOX
Cefuroxime	FUR, CXM	CXM
Ceftazidime	CAZ, CTZ, CF	CAZ
Cefoperazone	CFP	CFP
Cefaclor	CEC, CFC	CFC
Cefpodoxime	CPD	CPD
Monobactams		
Aztreonam	ATM, AZT, AM	ATM
Ertapenem	ETP	ETP
Imipenem	IPM, IMP, IMI	IMI
Meropenem	MEM	MEM
Aminoglycosides		
Gentamicin	GM, G, CN, GE, Gm, GN	GEN
Tobramycin	TOB, To	TOB
Amikacin	AMI, AM, AMK, AN, Ak	AMK
Kanamycin	KAN, K	KAN
Streptomycin	S, STR, SM, EST	STR
Fluoroquinolones		
Ciprofloxacin	CIP, Cp, CI, CPF, CPX	CIP
Levofloxacin	Lvx	Lvx
Ofloxacin	OFX	OFX
Norfloxacin	NOR	NOR
Quinolones		
Nalidixic acid	NA, NAL, Nx, N	NAL
Folate pathway inhibitors		
Trimethoprim- sulfamethoxazole	SXT, COT, ST, TMP—SLF, TS	SXT
Sulfonamides	SSS, SMX, sul, SUL, SMX	SUL
Trimethoprim	TMP, TRIM, TP, W	TMP
Phenicols		
Chloramphenicol	C, CHL, CM, CLF, CLO, CRO	CHL
Nitrofurans		
Nitrofurantoin	FT, NIT	NIT
Tetracyclines		
Tetracycline	TE, TET, T, TCY	TET
