# Supplementary material for: Antimicrobial Resistance of Non-Typhoid Salmonella in Meat and Meat Products
Source: Foods. 2021 Jul 27;10(8):1731. doi: 10.3390/foods10081731 (PMC8392175; doi:10.3390/foods10081731)
Supplement: Supplementary file 1 [file foods-10-01731-s001.zip › foods-1203532-supplementary.pdf]

Supplementary material

Antimicrobial resistance of Non-Typhoid Salmonella in meat and meat products

Sandra M. Rincón-Gamboa[1,2], Raúl A. Poutou-Piñales[2]*, Ana K Carrascal-Camacho[1]

[1] Laboratorio de Microbiología de Alimentos. Grupo de Biotecnología Ambiental e Industrial (GBAI). Departamento de Microbiología, Facultad de Ciencias, Pontificia Universidad Javeriana. Bogotá D.C., Colombia.

[2] Laboratorio Biotecnología Molecular. Grupo de Biotecnología Ambiental e Industrial (GBAI). Departamento de Microbiología, Facultad de Ciencias, Pontificia Universidad Javeriana. Bogotá D.C., Colombia.

*Corresponding Author
Raúl A. Poutou-Piñales, Ph.D.
Professor
*E-mail: rpoutou@javeriana.edu.co
Pontificia Universidad Javeriana, Bogotá, D.C., Colombia
Facultad de Ciencias
Departamento de Microbiología. Grupo de Biotecnología Ambiental e Industrial (GBAI).
Laboratorio de Biotecnología Molecular
Carrera 7ma No 43-82, Edificio 50 Lab. 124
Código Postal: Bogotá 110-23
Fax: 57-1 320 83 20 ext: 4021

Table S1.

Serovars that were reported in 1 to 3 studies.

Serovars reported in a study	
**Species**	***Salmonella* Serovars**
Poultry	Aberdeen, Altona, Arapahoe, Assine, Athinai, Augustenborg, Baiboukoum, Ball, Banana, Bardo, Bellevue, Berta, Bessi, Blegdam, Bonn,Bradford, Bsilla, Chartres, Chinco, Colindale, Cuckmere, Dabou, Dessau, Djugu, Duesseldorf, Duisburg, Duval, Edinburg, Fillmore, Gallinarum, Gatuni, Glostrup, Gueuletapee, Haardt, Hidalgo, Hoboken, Hoghton, Hull, I:4,5,12:i:-, I:6,7,14:K:-, I:6,8:-:1,2, I:ROUGH-O:gms:-, I:ROUGH-O:i:1,2, I:ROUGH-O:i:z6, I:ROUGH-O:r:1,2, I:ROUGH-O:z10:enx, II, Isangui, K:1,5, Kaapstad, Kallo, Kiel, Kingston, Kouka, Kunduchi, Leer, Lindenburg, Lome, Lome (Vi+), Maastricht, Magherafelt, Manhattan, Massenya, Minnesota, Nchanga, Newlands, Nitra, Ouakam, Poona, Potsdam, Quakam, Regent, Remo, Rough (b:1,2), Rough (g,m:-), Rumford, Sangera, Schleissheim, Schwerin, Shubra, Subsp. I, 4,5,12:1,2:-, Subsp. I, 4,5,12:g,m:-, Subsp. I,w, z28:1,2, Subsp.r:1,2, Tado, Tinda, Tokoin, Ughelli, Uppsala, Virginia, Vitkin, Wagenia, Wangat, Warragul, Westhampton, Winterthur, Yovokome.
Poultry and bovines	Brunei, Lockleaze
Poultry and pork	Emek
Poultry, bovines, and pork	Amsterdam, Apeyeme, I(1),4,(5),12:i,-, I4, 5,12:I:-, Typhimurium monofasica
Bovines	16:I,v:-, 4,12:nomotil, Cannstatt, Grupo 18, Grupo B, Grupo C1, Grupo E1, Grupo E1, Monofasica, Grupo F, Grupo G1, Grupo G2, Grupo G2 Monofasica, Grupo II, Javiana, Lomita, Molade, Oranienburg, Urbana, Viele, Azteca.
Bovines and pork	Bahrenfeld, Hvittingfodd
Pork	4,5,12:I:-, 4,5,12:-:1,7, 4:b:-, Alachua, Carrau, Coeln, India, Krefeld, Lagos, Limere, O:4,5, O:6,7, O:9:12, Rough, Wandsworth, Livingstone
**Serovars reported in two studies**	
**Species**	***Salmonella* Serovars**
Poultry	Abony, Blocley, Brancaster, Essen, Gaminara, Hillingdon, I:4,12:i:-, Kiambu, Litchfield, Rubislaw, Tshiongwe
Poultry and bovines	Dublin, Richmond

Poultry, bovines, and pork	Pomona, Bousso IV
Poultry and pork	Johannesburg
Bovines and pork	Sinstorf, Adelaide, Cholerasuis
**Pork**	**Chailey, Goldcoast**
**Serovars reported in three studies**	
**Species**	***Salmonella* Serovars**
Poultry and bovines	Haifa, Havana, Muenchen
Poultry, bovines, and pork	Cerro, Orion, Worthington

Table S2.
The concentration of antimicrobial agent used in the antimicrobial susceptibility test by MIC

Antimicrobial agent	Automated methods				CLSI reference method	
	Trek diagnostics, Westlake, OH	Reference	Phoenix NMIC/ID-121	Reference	Agar dilution method	Reference
AMP	1-32	[1-5]	4-16	[6, 7]	0.5-64	[8]
					4-32	[9]
AMC	1-32/0.5-16	[2-5]	4/2-16/8		4/2-32/16	
CEP	2-32	[3, 5]	1-16			
CTX			4-32		0.06-8	[8]
					4-64	[9]
CRO	0.25-64	[1-5]	2-32		2-64	
FOX	0.5-16	[3, 5]	4-16		4-32	
	0.5-32	[1, 2, 4]				
GEN	0.25-16	[1-5]	2-8		0.25-0.32	[8]
					2-16	[9]
AMK	0.5-4	[3, 5]	8-32		8-64	
	0.5-64	[1]				
KAN	8-64	[1-5]			4-64	
STR	32-64				32-64	
					2-512	[8]
CIP	0.015-4	[2, 3]	0.5-2		0.008-8	
	0.5-4				1-8	[9]
NAL	0.5-32				2-512	[8]
					4-32	[9]
SXT	0.12/4-2.38/76		0.5/9.5-2/38		0.5/9.5-4/76	
SUL	16-512	[3, 5]			64-512	
TMP					0.25-32	[8]
CHL	2-32	[1, 3-5]			2-256	
					4-32	[9]

TET	4-32		2-8		0.5-64	[8]
					2-16	[9]

Table S3.

Multi-resistance patterns reported once using MIC technique.

Ref.	Pattern	Serovar/ (Total serovar)	Total isolates with the pattern	% Isolates with the pattern
[5]	AMC FOX GEN KAN STR	Seftenberg (1)	1	100
[10]	AMP AMC CEP CRO FOX GEN KAN STR SUL TET	Bredney (1)	1	100
[5]	AMP AMC CEP FOX GEN KAN STR	Ouakam (2)	1	50
	AMP AMC CEP FOX GEN KAN STR NAL	Anatum (3)	1	33
	AMP AMC CEP FOX SUL	Litchfield (1)	1	100
	AMP AMC CEP FOX SUL TET	Agona (2)	2	100
	AMP AMC CEP GEN	Enteritidis (10)	1	10
	AMP AMC CEP GEN KAN STR NAL	Anatum (3)	1	33
	AMP AMC CEP GEN STR TET	Saintpaul (1)	1	100
[1]	AMP AMC CRO FOX GEN STR SXT TET	Anatum (3)	1	33
	AMP AMC CRO FOX KAN STR SXT CHL TET	Heidelberg (9)	1	11
	AMP AMC CRO FOX KAN SXT TET	Heidelberg (9)	2	22
	AMP AMC CRO FOX STR TET	Kentucky (4)	2	50
	AMP AMC CRO FOX TET	Kentucky (4)	2	50
[11] *	AMP AMC CTX CRO FOX ATM GEN KAN STR CIP NAL SXT CHL TET	Typhimurium (20)	1	5
	AMP AMC CTX CRO FOX CPD ATM GEN KAN STR NAL SXT CHL TET	Typhimurium (20)	1	5
	AMP AMC CTX CRO FOX CPD ATM GEN KAN STR SXT CHLTET	Enteritidis (10)	2	20
	AMP AMC CTX CRO FOX CPD ATM GEN KAN STR SXT TET	Typhimurium (20)	1	5
	AMP AMC CTX CRO FOX CPD ATM KAN STR CIP NAL SXT CHL TET	Typhimurium (20)	1	5
	AMP AMC CTX CRO FOX CPD GEN KAN STR SXT TET	Typhimurium (20)	1	5
	AMP AMC CTX FOX ATM GEN KAN STR CIP NAL SXT CHL TET	Enteritidis (10)	1	10
	AMP AMC CTX FOX ATM KAN STR CIP NAL SXT CHL TET	Typhimurium (20)	1	5
	AMP AMC CTX FOX KAN STR NAL SXT CHL TET	Typhimurium (20)	1	5
[5]	AMP AMC FOX GEN KAN STR TET	Ouakam (2)	1	50
[11] *	AMP ATM KAN STR	Enteritidis (10)	1	10
	AMP ATM KAN STR SXT CHL TET	Typhimurium (20)	1	5
[11] *	AMP CTX FOX ATM GEN KAN STR CIP NAL SXT CHL	Enteritidis (10)	1	10

	AMP CTX FOX ATM GEN KAN STR CIP NAL SXT TET	Typhimurium (20)	1	5
	AMP CTX FOX ATM GEN KAN STR NAL SXT CHL	Typhimurium (20)	1	5
	AMP CTX FOX ATM GEN KAN STR SXT TET	Infantis (6)	1	17
	AMP FOX ATM GEN KAN STR CIP NAL SXT CHL TET	Typhimurium (20)	2	9
	AMP FOX ATM KAN STR CIP NAL SXT TET	Typhimurium (20)	1	5
	AMP FOX ATM KAN STR SXT TET	Enteritidis (10)	1	10
	AMP FOX GEN KAN STR NAL SXT TET	Enteritidis (10)	1	10
	AMP FOX KAN STR SXT TET	Enteritidis (10)	1	10
	AMP FOX STR CIP NAL SXT TET	Typhimurium (20)	1	5
	AMP GEN KAN STR NAL SXT TET	Typhimurium (20)	4	18
	AMP GEN KAN STR SXT TET	Enteritidis (10)	1	10
[10]	AMP GEN STR SXT SUL TET	Brandenburg (1)	1	100
[11] *	AMP KAN STR SXT CHL TET	Infantis (6)	1	17
[1]	AMP KAN TET	I:ROUGH-O:i:1,2 (1)	1	100
	AMP STR CHL TET	Typhimurium (20)	1	5
[11] *	AMP STR CIP NAL CHL TET	Infantis (6)	1	17
	AMP STR SXT CHL	Enteritidis (10)	1	10
[1]	AMP STR TET	Hadar (1)	1	100
[3]	GEN KAN STR SUL TET	Heidelberg (9)	6	67
[11] *	GEN STR CIP NAL SXT CHL TET	Typhimurium (20)	1	5
[12]	STR CIP NAL SUL TET	Infantis (6)	3	50

.

Table S4.
Calculated antimicrobial susceptibility frequency rates of Salmonella serovars using the disc diffusion technique.

Ref.	Serovar	Isolates number	AMC	SAM	CFZ	CEP	CTX	CRO	FOX	CAZ	FEP	ATM	IMI	AMK
[13]	Kentucky	27.0				40.7			37.0					
	Muenster	12.0				41.7			41.7					
	Enteritidis	6.0				33.3			33.3					
	Hadar	6.0												
	Mbandaka	3.0												
	Schwarzengrund	2.0												
	Corvallis	1.0				100			100					
[14]	Enteritidis	27.0		40.7	48.1	77.8								
	Typhimurium	3.0				100								
[15]	Thompson	54.0	5.6											
	Enteritidis	25.0												
	Typhimurium	14.0												
	Hadar	6.0												
[16]	Typhimurium	5.0	80,0	80,0				20,0						
	Derby	1.0	100											
[17]	Typhimurium	84.0	2.4				9.5			2.4	6.0		1.2	1.2
	Rissen	81.0					1.2			1.2				
	Derby	75.0	2.7				5.3				4.0			1.3
	London	57.0											3.5	
	Agona	56.0					5.4			1.8				1.8
	Corvallis	46.0					8.7			4.3	2.2			
	Kentucky	38.0					10.5							2.6
	Mbandaka	34.0					2.9			2.9				2.9
[18]	Derby	79.0	20.3		20.3			1.3	5.1	7.6	1.3			2.5

Typhimurium	22.0	18.2	18.2	4.5	4.5	4.5	4.5			4.5
London	20.0	25.0	25.0	5.0	5.0	5.0	10.0	10.0		
Rissen	18.0	27.8	27.8				5.6			
Enteritidis	7.0	71.4	71.4			57.1	71.4	28.6		14.3
Indiana	4.0	75.0	75.0	100	100	50.0	50.0	100	100	75.0
Corvallis	3.0									
Stanley	3.0					33.3	33.3			
Anatum	3.0	33.3	33.3			33.3	66.7			
Thompson	3.0	33.3	33.3	33.3	33.3		33.3			

Table S5

Reported resistance percentages using disc diffusion method

Reference	Serovar (s)	Nº isolates	GEN		STR		SXT		TMP		SUL		TET		AMP		CEP		FOX	
			N	% C	N	% C	N	% C	N	% C	N	% C	N	% C	N	% C	N	% C	N	% C
	Brancaster	8		0	8	100	7	88	7	88	8	100	8	100		0		0		0
	Bredeney	4	1	25		0		0	3	75		0	1	25	2	50	3	75	3	75
	Johannesburg	4		0		0		0		0	1	25	4	100		0		0		0
	Vitkin	3		0		0	3	100	3	100	3	100	3	100	3	100		0		0
[13]	Chester	3		0		0	1	33	1	33	1	33		0		0		0		0
	Duval	1		0		0		0		0		0	1	100		0		0		0
	Hull	1		0		0		0		0		0	1	100	1	100	1	100	1	100
	Tado	1		0		0		0		0		0	1	100		0	1	100		0
	Tshiongwe	1		0		0		0		0		0	1	100	1	100	1	100		0

Reference	Serovar (s)	Nº isolates	STR			NAL			SXT			SUL			CHL			TET		
			N	% C	% R	N	% C	% R	N	% C	% R	N	% C	% R	N	% C	% R	N	% C	% R
[19]	Brandenburg	14	1	7	7	5	36	36	12	86	86	12	86	86	1	7	7	13	93	93

Reference	Serovar (s)	Nº isolates	STR			NAL			TMP			CHL			TET		
			N	% C	% R	N	% C	% R	N	% C	% R	N	% C	% R	N	% C	% R
[20]	Virginia	3	1	33	33	3	100	100	3	100	100	1	33	33	3	100	100

Reference	Serovar (s)	Nº isolates	STR			NAL			TET		
			N	% C	% R	N	% C	% R	N	% C	% R
	Montevideo	6	4	67	67	6	100	100	1	17	17
[14]	Reading	1		0		1	100	100		0	
	Senftenberg	1	1	100	100	1	100	100		0	

Reference	Serovar (s)	Nº isolates	SXT			TMP			CHL			TET			AMP		
			N	% C	% R	N	% C	% R	N	% C	% R	N	% C	% R	N	% C	% R

[21]	Dabou	40	6	15	15	6	15	15	4	10	10	25	63	#	6	15	15

Reference	Serovar (s)	Nº isolates	GEN			KAN			STR			CIP			NAL			CHL			AMP		
			N	% C	% R	N	% C	% R	N	% C	% R	N	% C	% R	N	% C	% R	N	% C	% R	N	% C	% R
[22]	Haifa	10	1	10	10	3	30	30	6	60	60	6	60	60	5	50	100	4	40	40	8	80	90
	Virchow	5		0		1	20	10	3	60	60	2	40	40	2	40	100	2	40	40	5	100	100

Reference	Serovar (s)	Nº isolates	KAN			STR			NAL			TMP			CHL			TET			AMP			AMC		
			N	% C	% R	N	% C	% R	N	% C	% R	N	% C	% R	N	% C	% R	N	% C	% R	N	% C	% R	N	% C	% R
[15]	Newport	8	3	38	38	4	50	50	7	88	88	1	13	13	1	13	13	8	100	100	1	13	13	2	25	25

Reference	Serovar (s)	Nº isolates	GEN			STR			NAL			SXT			CHL			TET			AMP			AMC			SAM		
			N	% C	% R	N	% C	% R	N	% C	% R	N	% C	% R	N	% C	% R	N	% C	% R	N	% C	% R	N	% C	% R	N	% C	% R
[16]	Kiel	1		0			0			0			0			0		1	100	100	1	100	100	1	100	100		0	
	Rubislaw	1	1	100	100	1	100	100	1	100	100	1	100	100	1	100	100	1	100	100	1	100	100	1	100	100	1	100	100

Reference	Serovar (s)	Nº isolates	NAL		TET		AMP	
			N	% C	N	% C	N	% C
[23]	S. 4,5,12:i:-	2	2	100	2	100	2	100
	Braenderup	3	3	100	1	33		0
	Bareily	1	1	100		0	1	100
	Give	1	1	100		0		0
	Saintpaul	1		0		0	1	100

Reference	Serovar (s)	Nº isolates	GEN			AMK			STR			CIP			OFX			NAL		
			N	% C	% R	N	% C	% R	N	% C	% R	N	% C	% R	N	% C	% R	N	% C	% R
[17]	Typhimurium	84	13	15	16	1	1	1	50	60	60	11	13	13	25	30	30	25	30	30
	Rissen	81	2	2	3		0		16	20	20	1	1	1	7	9	9	6	7	7
	Derby	75	27	36	36	1	1	1	27	36	36	25	33	33	32	43	43	30	40	40
	London	57	33	58	58		0		39	68	68		0		9	16	16	1	2	2
	Agona	56		0		1	2	2	9	16	16	1	2	2	51	91	91	11	20	20
	Corvallis	46		0			0		8	17	17	8	17	17	40	87	87	20	43	44
	Kentucky	38	3	8	8	1	3	3	5	13	14	11	29	31	25	66	69	11	29	31

	Mbandaka	34	1	3	3	1	3	3	25	74	74	1	3	3	21	62	62	1	3	3

Reference	Serovar (s)	Nº isolates	SXT			SUL			CHL			TET			AMP			AMC			CTX			CAZ			FEP			IMI		
			N	% C	% R	N	% C	% R	N	% C	% R	N	% C	% R	N	% C	% R	N	% C	% R	N	% C	% R	N	% C	% R	N	% C	% R	N	% C	% R
[17]	Typhimurium	84	23	27	27	75	89	89	34	40	41	69	82	82	70	83	83	2	2	2	8	10	10	2	2	2	5	6	6	1	1	1
	Rissen	81	63	78	78	63	78	78	7	9	9	77	95	95	63	78	78		0		1	1	1	1	1	1		0			0	
	Derby	75	32	43	43	69	92	92	33	44	44	61	81	81	42	56	56	2	3	3	4	5	5		0		3	4	4		0	
	London	57	40	70	70	44	77	77	24	42	42	49	86	86	35	61	61		0			0			0			0		2	4	4
	Agona	56	10	18	18	23	41	41	50	89	89	27	48	48	9	16	16		0		3	5	5	1	2	2		0			0	
	Corvallis	46	8	17	17	34	74	74	24	52	52	37	80	80	8	17	17		0		4	9	9	2	4	4	1	2	2		0	
	Kentucky	38	26	68	72	35	92	97	30	79	83	33	87	92	8	21	22		0		4	11	11		0			0			0	
	Mbandaka	34	2	6	6	34	100	100		0		33	97	97					0		1	3	3	1	3	3		0			0	

Reference	Serovar (s)	Nº isolates	GEN		AMK		KAN		STR		CIP		NAL		SXT		CHL		TET		AMP		AMC	
			N	% C	N	% C	N	% C	N	% C	N	% C	N	% C	N	% C	N	% C	N	% C	N	% C	N	% C
[18]	1,4,[5],12:i:-	15	3	20	0	0	4	27	11	73	4	27	6	40	4	27	5	33	14	93	14	93	5	33
	Meleagridis	4	0	0	1	25	0	0	1	25	0	0	0	0	4	100	1	25	4	100	1	25	1	25
	Stanley	3	0	0	0	0	0	0	1	33	1	33	0	0	0	0	1	33	1	33	0	0	0	0
	Infantis	3	0	0	1	33	1	33	1	33	0	0	3	100	1	33	1	33	1	33	2	67	2	67

Reference	Serovar (s)	Nº isolates	CFZ		CTX		CRO		FOX		CAZ		FEP		ATM		IMI	
			N	% C	N	% C	N	% C	N	% C	N	% C	N	% C	N	% C	N	% C
[18]	1,4,[5],12:i:-	15	5	33	0	0	1	7	0	0	0	0	1	7	0	0	2	13
	Meleagridis	4	1	25	1	25	1	25	0	0	1	25	2	50	1	25	0	0
	Stanley	3	0	0	0	0	0	0	1	33	1	33	0	0	0	0	0	0
	Infantis	3	2	67	2	67	2	67	2	67	2	67	2	67	2	67	0	0

Yellow boxes indicate differences in reported resistance prevalence percentages by the authors and those calculated in this study.

Table S6.
Multiresistance patterns described only one for each serovar using the disk diffusion technique.

Reference	Multiresistance Patterns	Serovar (Total isolates)	Total isolates with multiresistance pattern	Multiresistance pattern (% Frequency)
[14]	AMC CEP NAL SXT	Enteritidis (102)	1	1
	AMC CEP STR NAL	Montevideo (2)	1	50
	AMC CEP STR NAL	Senftenberg (3)	1	33
	AMC CFZ CEP GEN KAN STR NAL TET	Enteritidis (113)	1	1
	AMC CFZ CEP GEN STR NAL TET	Enteritidis (113)	2	2
[15] *	AMC NAL TET	Newport (7)	2	29
[14]	AMC SAM CEP STR NAL CHL	Enteritidis (113)	1	1
	AMC SAM CFZ CEP GEN KAN STR NAL TET	Enteritidis (113)	8	8
	AMC SAM CFZ CEP GEN STR NAL TET	Enteritidis (113)	1	1
	AMC SAM CFZ STR NAL CHL	Enteritidis (113)	1	1
[16]*	AMC SAM GEN CIP NAL SXT CHL TET	Typhimurium (114)	1	1
	AMC SAM GEN STR SXT TET	Typhimurium (114)	1	1
[15]*	AMC STR NAL SXT TMP TET	Thompson (16)	1	6
[16]*	AMC STR TET	Derby (47)	1	2
[24]*	AMK CHL CIP NOR SXT GEN AMP AMC	Thompson (16)	1	6
[25] *	AMK CHL CIP SXT AMP AMC	Newport (7)	1	14
	AMK CHL CIP TET NOR AMP	Kentucky (20)	1	5
	AMK CHL CIP TET NOR STR GEN AMP	Typhimurium (114)	1	1
	AMK CHL CIP TET NOR STR GEN NAL AMP	Derby (47)	1	2
	AMK CHL CIP TET NOR STR NAL AMP	Derby (47)	1	2
	AMK CHL CIP TET NOR SXT GEN AMP	Kentucky (20)	1	5
	AMK CHL CIP TET NOR SXT GEN AMP	Kentucky (20)	1	5
	AMK CHL CIP TET NOR SXT STR	Derby (47)	2	4
	AMK CHL CIP TET NOR SXT STR AMP	Derby (47)	1	2
	AMK CHL CIP TET NOR SXT STR GEN AMP	Derby (47)	1	2
	AMK CHL CIP TET NOR SXT STR GEN AMP	Meleagridis (6)	1	17
	AMK CHL CIP TET NOR SXT STR GEN AMP	Typhimurium (114)	1	1
	AMK CHL CIP TET NOR SXT STR GEN NAL	Derby (47)	1	2
	AMK CHL CIP TET NOR SXT STR NAL	Typhimurium (114)	1	1
	AMK CHL CIP TET SXT AMP	Kentucky (20)	1	5
	AMK CHL CIP TET SXT STR GEN AMP	Meleagridis (6)	1	17
	AMK CHL CIP TET SXT STR NAL AMP	Derby (47)	1	2
	AMK CIP AMP	Typhimurium (114)	2	2
	AMK CIP NOR SXT AMP	Enteritidis (113)	1	1
	AMK CIP STR AMP	Enteritidis (113)	1	1
	AMK CIP STR GEN	Tennessee (2)	1	50
	AMK CIP TET	Derby (47)	1	2
	AMK CIP TET GEN	Derby (47)	1	2
	AMK CIP TET NOR STR GEN NAL	Derby (47)	1	2
	AMK CIP TET NOR SXT STR GEN AMP AMC	Rissen (9)	1	11
	AMK CIP TET STR AMP	Enteritidis (113)	1	1
	AMK CIP TET STR GEN	Corvallis (1)	1	100
	AMK CIP TET STR GEN	Tennessee (2)	1	50

	AMK CIP TET STR GEN AMP	Meleagridis (6)	1	17
	AMK CIP TET STR GEN AMP	Typhimurium (114)	1	1
	AMK CIP TET SXT	Meleagridis (6)	1	17
	AMK CIP TET SXT AMP	Enteritidis (113)	1	1
	AMK CIP TET SXT AMP	Rissen (9)	1	11
	AMK CIP TET SXT STR AMP	Enteritidis (113)	1	1
	AMK CIP TET SXT STR GEN	Derby (47)	2	4
	AMK CIP TET SXT STR GEN AMP	Rissen (9)	1	11
	AMK SXT CIP STR AMP AMC	Newport (7)	1	14
	AMK TET CIP	Derby (47)	1	2
	AMK TET STR GEN AMP	Derby (47)	1	2
	AMK TET SXT AMP	Rissen (9)	1	11
	AMK TET SXT STR GEN	Derby (47)	1	2
[26]	AMP AMC AMK STR NAL	Winterthur (2)	1	50.00
[27]	AMP AMC CEP STR NAL SXT CHL TET	Enteritidis (113)	1	1
[28]*	AMP AMC CRO CIP NAL SXT CHL TET	Albany (6)	1	17
	AMP AMC CRO GEN CHL	Saintpaul (9)	1	11
[26]	AMP AMC CRO STR SXT	Winterthur (2)	1	50.00
[24]*	AMP AMC CTX CRO CAZ CPD STR NAL SXT TET	Typhimurium (114)	1	1
[28]*	AMP AMC NAL SXT CHL TET	Albany (6)	1	17
	AMP AMC NAL SXT CHL TET	Brancaster (16)	3	19
[16]*	AMP AMC SAM GEN STR NAL SXT CHL TET	Rubislaw (1)	1	100
	AMP AMC SAM GEN STR NAL SXT CHL TET	Typhimurium (114)	2	2
[28]*	AMP AMC SXT CHL TET	Brancaster (16)	1	6
[16]*	AMP AMC TET	Kiel (1)	1	100.00
[28]*	AMP ATM GEN KAN STR SXT CHL TET	Infantis (1)	1	100
[26]	AMP CAZ STR TET	Typhimurium (114)	1	1
	AMP CEP AMK	Typhimurium (114)	1	1
	AMP CEP CRO CAZ GEN KAN STR NAL TET AMP	Westhampton (1)	1	100.00
[13]	AMP CEP FOX STR	Kentucky (20)	1	5
	AMP CEP FOX STR NAL TET	Enteritidis (113)	2	2
	AMP CEP FOX STR SXT SUL TMP TET	Muenster (8)	1	13
	AMP CEP FOX SXT	Kentucky (20)	1	5
	AMP CEP FOX SXT SUL TMP	Muenster (8)	2	25
	AMP CEP FOX SXT SUL TMP TET	Muenster (8)	2	25
	AMP CEP FOX SXT SUL TMP TET	Kentucky (20)	1	5
	AMP CEP FOX TET	Hull (1)	1	100.00
	AMP CEP FOX TMP	Bredeney (5)	1	20
	AMP CEP FOX TMP	Kentucky (20)	1	5
	AMP CEP FOX TMP TET	Kentucky (20)	1	5
[29] *	AMP CEP GEN STR CHL TET	Typhimurium (114)	1	1
	AMP CEP GEN STR SXT CHL TET	Anatum (5)	2	40
[30]	AMP CEP KAN STR NAL TET	Hadar (16)	1	6
[31]*	AMP CEP STR CIP SXT SUL CHL TET	Typhimurium (114)	1	1
	AMP CEP STR SXT SUL CHL	Typhimurium (114)	1	1
[30]	AMP CEP STR TET	Hadar (16)	1	6
[13]	AMP CEP TET	Kentucky (20)	1	5
	AMP CEP TET	Tado (1)	1	100

[26]	AMP CFZ CAZ IMI KAN STR NAL TET	Typhimurium (114)	1	1
[32] *	AMP CFZ CEP CTX GEN STR TET	Lome (Vi+) (1)	1	100.00
[33] *	AMP CFZ CIP SXT CHL	Typhimurium (114)	2	2
[34]	AMP CFZ CRO STR NAL SXT CHL TET	Derby (47)	1	2
[35]*	AMP CFZ CTX CRO KAN CIP NAL SXT CHL TET	Typhimurium (114)	1	1
[34]	AMP CFZ NAL SXT CHL TET	Meleagridis (6)	1	17
	AMP CFZ NAL TET	Derby (47)	1	2
	AMP CHL TET	Derby (47)	1	2
[30]	AMP CIP NAL TET	Typhimurium (114)	1	1
[26]	AMP CRO CAZ GEN AMK	Typhimurium (114)	1	1
[17] *	AMP CTX GEN CIP OFX NAL SUL CHL	Kentucky (20)	1	5
	AMP CTX GEN CIP OFX NAL SXT CHL TET	Schwarzengrund (3)	1	33
	AMP CTX GEN CIP OFX NAL SXT SUL CHL TET	Kentucky (20)	1	5
	AMP CTX GEN STR CIP OFX NAL SXT SUL CHL	Indiana (8)	1	12.50
	AMP CTX GEN STR CIP OFX NAL SXT SUL CHL TET	Indiana (8)	1	12.50
	AMP CTX GEN STR OFX SXT SUL CHL TET	Litchfield (4)	2	50.00
	AMP CTX GEN STR SXT SUL CHL TET	Typhimurium (114)	1	1
[23] *	AMP CTX NAL SXT CHL TET	Rissen (9)	1	11
[17] *	AMP CTX OFX SUL CHL	Kentucky (20)	1	5
	AMP CTX STR SUL TET	Typhimurium (114)	1	1
	AMP FEP CTX CAZ GEN AMK CIP OFX NAL SXT SUL CHL TET	Indiana (8)	1	12.50
	AMP FEP CTX CAZ GEN CIP OFX NAL SXT SUL CHL TET	Indiana (8)	1	12.50
	AMP FEP CTX CAZ GEN STR CIP OFX NAL SXT SUL CHL TET	Indiana (8)	1	12.50
	AMP FEP CTX CAZ GEN STR OFX SXT CHL TET	Litchfield (4)	1	25.00
	AMP FEP CTX CAZ GEN STR SXT SUL	Litchfield (4)	1	25.00
	AMP FEP CTX CAZ STR OFX SUL TET	Typhimurium (114)	1	1
	AMP FEP CTX GEN AMK STR CIP OFX NAL SXT SUL CHL TET	Indiana (8)	2	25.00
	AMP FEP CTX NAL TET	Derby (47)	2	4
	AMP FEP CTX STR OFX NAL SUL CHL TET	Typhimurium (114)	1	1
	AMP FEP CTX STR OFX SXT SUL CHL TET	Typhimurium (114)	1	1
[34]	AMP GEN KAN CIP NAL SXT CHL TET	Derby (47)	1	2
[35]*	AMP GEN KAN CIP NOR NAL CHL TET	Derby (47)	1	2
	AMP GEN KAN NAL SXT CHL	Typhimurium (114)	2	2
[34]	AMP GEN KAN NAL SXT CHL TET	Meleagridis (6)	1	17
[36] *	AMP GEN KAN STR CIP NOR NAL SXT CHL	Typhimurium (114)	2	2
[30]	AMP GEN KAN STR NALTET	Blockey (2)	1	50.00
[37]	AMP GEN KAN STR SUL TMP TET	Typhimurium (114)	1	1
[34]	AMP GEN KAN STR SXT CHL TET	Typhimurium (114)	1	1

	AMP GEN KAN SXT TET	Typhimurium (114)	1	1
[26]	AMP GEN NAL SXT	Enteritidis (113)	1	1
[28]*	AMP GEN NAL SXT CHL TET	Albany (6)	1	17
	AMP GEN NAL SXT CHL TET	Brancaster (16)	4	25
[38]	AMP GEN STR CIP SXT CHL TET	Indiana (8)	1	12.50
[26]	AMP GEN STR NAL	Enteritidis (113)	1	1
[35]*	AMP GEN STR NAL CHL TET	London (5)	1	20
[34]	AMP GEN STR NAL SXT CHL TET	Newport (7)	1	14
	AMP GEN STR NAL SXT CHL TET	Derby (47)	1	2
[35]*	AMP GEN STR OFX NOR NAL SXT CHL TET	London (5)	1	20
[30]	AMP GEN STR SXT TET	Hadar (16)	1	6
[37]	AMP GEN SUL CHL	Havana (2)	1	50
[28]*	AMP GEN SXT CHL TET	Saintpaul (9)	5	56
	AMP GEN SXT CHL TET	Brancaster (16)	1	6
[39]*	AMP GEN SXT TET	Bredeney (5)	1	20
	AMP GEN SXT TET	Derby (47)	6	13
	AMP GEN SXT TET	Typhimurium (114)	3	3
[34]	AMP KAN NAL SXT CHL TET	Typhimurium (114)	1	1
[15]*	AMP KAN NAL TET	Hadar (16)	1	6
[36] *	AMP KAN STR CIP NOR NAL SXT CHL	Enteritidis (113)	4	4
	AMP KAN STR CIP NOR NAL SXT CHL	Typhimurium (114)	10	9
[34]	AMP KAN STR NAL SXT TET	Give (3)	1	33
[15]*	AMP KAN STR NAL SXT TMP TET	Thompson (16)	1	6
[30]	AMP KAN STR NAL TET	Blockey (2)	1	50.00
[26]	AMP KAN STR SXT TET	Enteritidis (113)	1	1
[34]	AMP KAN SXT CHL TET	Muenster (8)	1	13
[37]	AMP NAL SUL TMP CHL TET	Albany (6)	1	17
[23] *	AMP NOR NAL SXT CHL TET	Albany (6)	2	33
[34]	AMP OFX NAL	Derby (47)	2	4
[32] *	AMP STR CHL	Enteritidis (113)	1	1
[36] *	AMP STR CIP NOR NAL CHL	Enteritidis (113)	8	8
	AMP STR CIP NOR NAL SXT CHL	Typhimurium (114)	8	7
[31]*	AMP STR CIP SXT SUL CHL TET	Enteritidis (113)	1	1
[29] *	AMP STR NAL CHL TET	Give (3)	1	33
[36] *	AMP STR NAL SXT	Enteritidis (113)	14	14
	AMP STR NAL SXT	Kentucky (20)	2	10
	AMP STR NAL SXT	Typhimurium (114)	12	11
	AMP STR NAL SXT	Virchow (6)	6	100
	AMP STR NAL SXT CHL	Enteritidis (113)	20	20
	AMP STR NAL SXT CHL	Typhimurium (114)	8	7
[34]	AMP STR NAL SXT CHL TET	Derby (47)	1	2
[36] *	AMP STR NOR NAL SXT CHL	Enteritidis (113)	8	8
	AMP STR NOR NAL SXT CHL	Typhimurium (114)	10	9
[34]	AMP STR OFX NAL SXT CHL TET	Derby (47)	3	6
Yu et al., (2014)*	AMP STR SUL CHL TET	Enteritidis (113)	1	1
[37]	AMP STR SUL TET	Anatum (5)	1	20
[29] *	AMP STR SXT CHL TET	Sintorf (1)	1	100.00
[31]*	AMP STR SXT SUL	Anatum (5)	1	20

	AMP STR SXT SUL CHL TET	Enteritidis (113)	1	1
[13]	AMP STR SXT SUL TMP TET	Hadar (16)	1	6
	AMP STR SXT SUL TMP TET	Muenster (8)	1	13
[30]	AMP STR SXT TMP TET	Bredeney (5)	1	20
[29] *	AMP STR TET	Typhimurium (114)	1	1
[33] *	AMP SXT CHL	Enteritidis (113)	2	2
[31]*	AMP SXT SUL TET	Saintpaul (9)	1	11
[13]	AMP SXT SUL TMP TET	Schwarzengrund (3)	2	67
	AMP SXT SUL TMP TET	Vitkin (3)	3	100
[39]*	AMP SXT TET	Typhimurium (114)	8	7
[31]*	AMP TZP STR SXT SUL CHL TET	Enteritidis (113)	1	1
	CAZ ATM AMK SUL CHL	London (5)	1	20
	CAZ ATM TOB AMK CIP SXT SUL	London (5)	1	20
[13]	CEP FOX GEN TMP TET	Bredeney (5)	1	20
	CEP FOX GEN TMP TET	Bredeney (5)	1	20
[14]	CEP NAL TET	Montevideo (2)	1	50
	CEP STR CHL	Reading (1)	1	100.00
[26]	CFZ STR NAL	Typhimurium (114)	1	1
[25] *	CHL CIP NOR SXT STR AMP	Enteritidis (113)	1	1
	CHL CIP NOR SXT STR AMP	Enteritidis (113)	1	1
	CHL CIP STR AMP	Enteritidis (113)	3	3
	CHL CIP TET NOR STR AMP	Enteritidis (113)	1	1
	CHL CIP TET NOR SXT STR AMP	Derby (47)	1	2
	CHL CIP TET SXT AMP	Kentucky (20)	1	5
	CHL TET SXT STR GEN AMP	London (5)	1	20
	CIP NAL CHL TET	Give (3)	1	33
	CIP STR AMP	Enteritidis (113)	1	1
	CIP SXT STR AMP	Enteritidis (113)	1	1
[31]*	CIP SXT SUL CHL	Derby (47)	1	2
	CIP SXT SUL TET	Cholerasuis (1)	1	100.00
[25] *	CIP TET STR AMP	Enteritidis (113)	1	1
	CIP TET STR AMP	Rissen (9)	1	11
	CIP TET STR GEN AMP	Rissen (9)	1	11
[31]*	CRO ATM CIP SXT SUL	Lomita (1)	1	100.00
	CRO CAZ CFP ATM STR CIP SXT SUL	Kentucky (20)	1	5
[16]*	CRO GEN STR CIP NAL SXT TET	Typhimurium (114)	1	1
[32] *	GEN STR CHL TET	Typhimurium (114)	1	1
[34]	GEN STR CHL TET	Typhimurium (114)	1	1
[39]*	GEN SXT TET	Derby (47)	3	6
	GEN SXT TET	Typhimurium (114)	1	1
[15]*	KAN NAL CHL TET	Newport (7)	1	14
	KAN NAL SXT TET	Typhimurium (114)	1	1
	KAN NAL TET	Typhimurium (114)	1	1
	KAN STR NAL SXT TMP TET	Hadar (16)	3	19
	KAN STR NAL SXT TMP TET	Thompson (16)	3	19
	KAN STR NAL SXT TET	Thompson (16)	2	13
	KAN STR NAL TMP TET	Thompson (16)	5	31
	NAL SXT CHL TET	Hadar (16)	1	6

	NAL SXT TMP TET	Enteritidis (113)	3	3
	NAL SXT TMP TET	Hadar (16)	1	6
	NAL SXT TMP TET	Typhimurium (114)	2	2
	NAL SXT TMP TET	Hadar (16)	1	6
	NAL SXT TMP TET	Typhimurium (114)	2	2
	NAL TMP TET	Thompson (16)	3	19
	NAL TMP TET	Typhimurium (114)	1	1
[32] *	STR CHL TET	Rissen (9)	1	11
[31]*	STR CIP SXT SUL	Senftenberg (3)	2	67
[29] *	STR NAL CHL TET	Anatum (5)	1	20
[37]	STR NAL SUL TET	Anatum	ND	ND
	STR NAL SUL TET	London	ND	ND
[29] *	STR NAL SXT CHL TET	Typhimurium (114)	3	3
	STR NAL SXT CHL TET	Rissen (9)	1	11
	STR NAL SXT CHL TET	Typhimurium (114)	3	3
[15]*	STR NAL SXT TMP TET	Enteritidis (113)	2	2
[30]	STR NAL TMP	Enteritidis (113)	1	1
	STR NAL TMP	Hadar (16)	3	19
[15]*	STR NAL TMP TET	Newport (7)	1	14
	STR NAL TMP TET	Typhimurium (114)	1	1
[35]*	STR NOR TET	Derby (47)	1	2
[31]*	STR SXT SUL CHL	Derby (47)	1	2
	STR SXT SUL TET	Saintpaul (9)	1	11
	STR SXT SUL TET	Derby (47)	1	2
[13]	STR SXT SUL TET	Kentucky (20)	1	5
	STR SXT SUL TMP TET	Brancaster (16)	7	44
	STR SXT SUL TMP TET	Hadar (16)	2	13
	STR SXT SUL TMP TET	Muenster (8)	1	13
	STR SXT SUL TMP TET	Kentucky (20)	2	10
[29] *	STR SXT TET	Havana (2)	1	50
	STR SXT TET	Saintpaul (9)	1	11
[38]	SXT CHL TET	Warragul (1)	1	100.00
[31]*	TZP CEP STR SXT SUL CHL TET	Derby (47)	1	2
	TZP CRO STR SXT SUL CHL TET	Derby (47)	1	2

References

1. Aslam, M.; Checkley, S.; Avery, B.; Chalmers, G.; Bohaychuk, V.; Gensler, G.; Reid-Smith, R.; Boerlin, P. Phenotypic and genetic characterization of antimicrobial resistance in Salmonella serovars isolated from retail meats in Alberta, Canada. *Food Microbiol.* **2012**, 32, 110-117.
2. Bosilevac, J.M.; Guerini, M.N.; Kalchayanand, N.; Koohmaraie, M. Prevalence and Characterization of Salmonellae in Commercial Ground Beef in the United States. **2009**, 75, 1892–1900.
3. Fakhr, M.K.; Sherwood, J.S.; Thorsness, J.; Logue, C.M. Molecular Characterization and Antibiotic Resistance Profiling of Salmonella Isolated from Retail Turkey Meat Products. *Fodborne Pathog. Dis.* **2006**, 3, 366-374.
4. MIkanatha, N.M.; Sandt, C.H.; Localio, A.R.; Tewari, D.; Rankin, S.C.; Whichard, J.M.; Altekruse, S.F.; Lautenbach, E.; Folster, J.P.; Russo, A.; Chiller, T.M.; Reynolds, S.M.; McDermott, P.F. Multidrug-Resistant Salmonella Isolates from Retail Chicken Meat Compared with Human Clinical Isolates. *Foodborne Pathog. Dis.* **2010**, 7, 929-934.
5. Gad, A.H.; Abo-Shama, U.H.; Harclerode, K.K.; Fakhr, M.K. Prevalence, Serotyping, Molecular Typing, and Antimicrobial Resistance of Salmonella Isolated From Conventional and Organic Retail Ground Poultry. *Front. Microbiol.* **2018**, 9, Article 2653.
6. Donado-Godoy, P.; Byrne, B.A.; Hume, M.; Leon, M.; Perez-Gutierrez, E.; Vives Flores, M.J.; Clavijo, V.; Holguin, A.; Romero-Zuniga, J.J.; Castellanos, R.; Tafur, M.; Smith, W.A. Molecular Characterization of *Salmonella* Paratyphi B dT+ and *Salmonella* Heidelberg from Poultry and Retail Chicken Meat in Colombia by Pulsed-Field Gel Electrophoresis. *J. Food Prot.* **2015**, 78, 802-807.
7. Donado-Godoy, P.; Clavijo, V.; León, M.; Arevalo, A.; Castellanos, R.; Bernal, J.; Tafur, M.A.; Ovalle, M.V.; Alali, W.Q.; Hume, M.; Romero-Zuñiga, J.J.; Walls, I.; Doyle, M.P. Counts, Serovars, and Antimicrobial Resistance Phenotypes of Salmonella on Raw Chicken Meat at Retail in Colombia. *J. Food Prot.* **2014**, 77, 227-235.
8. Clemente, L.; Manageiro, V.; Ferreira, E.; Jones-Dias, D.; Correia, I.; Themudo, P.; Albuquerque, T.; Caniça, M. Occurrence of extended-spectrum β-lactamases among isolates of *Salmonella enterica* subsp. enterica from food-producing animals and food products, in Portugal. *Int. J. Food Microbiol.* **2013**, 167, 221-228.
9. Yang, B.; Cui, Y.; Shi, C.; Wang, J.; Xia, X.; Xi, M.; Wang, X.; Meng, J.; Alali, W.Q.; Walls, I.; Doyle, M.P. Counts, Serotypes, and Antimicrobial Resistance of Salmonella Isolates on Retail Raw Poultry in the People's Republic of China. *J. Food Prot.* **2014**, 77, 894-902.
10. Cook, A.; Reid-Smith, R.; Irwin, R.; Mcewen, S.A.; Valdivieso-Garcia, A.; Ribble, C. Antimicrobial Resistance in Campylobacter, Salmonella, and *Escherichia coli* Isolated from Retail Turkey Meat from Southern Ontario, Canada. *J. Food Prot.* **2009**, 72, 473–481.
11. Ahmed, A.M.; Shimamoto, T.; Shimamoto, T. Characterization of integrons and resistance genes inmultidrug-resistant *Salmonella enterica* isolated from meat and dairy products in Egypt. *Int. J. Food Microbiol.* **2014**, 189, 39-44.
12. Tirziu, E.; Lazar, R.; Sala, C.; Nichita, I.; Morar, A.; Ere, M.; Imre, K. Salmonella in Raw Chicken Meat from the Romanian Seaside: Frequency of Isolation and Antibiotic Resistance. *J. Food Prot.* **2016**, 78, 1003-1006.
13. Bada-Alambedji, R.; Fofana, A.; Seydi, M.; Akakpo, A.J. Antimicrobial Resistance of Salmonella Isolated From Poultry Carcasses In Dakar (Senegal). *Braz. J. Microbiol.* **2006**, 37, 510-515.
14. Kim , M.-S.; Lim , T.-H.; Jang , J.-H.; Lee , D.-H.; Kim , B.-Y.; Kwon , J.-H.; Choi , S.-W.; Noh , J.-Y.; Hong , Y.-H.; Lee , S.-B.; Yang , S.-Y.; Lee , H.-J.; Lee , J.-B.; Park , S.-Y.; Choi , I.-S.; Song, C.-S. Prevalence and antimicrobial resistance of Salmonella species isolated from chicken meats produced by different integrated broiler operations in Korea. *Poult. Sci.* **2012**, 91, 2370–2375.
15. Sodagari, H.R.; Mashak, Z.; Ghadimianazar, A. Prevalence and antimicrobial resistance of Salmonella serotypes isolated from retail chicken meat and giblets in Iran. *J. Infect. Dev. Countr.* **2015**, 9, 463-469.
16. Gharieb, R.M.; Tartor, Y.H.; Khedr, M.H.E. Non□Typhoidal Salmonella in poultry meat and diarrhoeic patients: prevalence, antibiogram, virulotyping, molecular detection and sequencing of class I integrons in multidrug resistant strains. *Gut Pathog.* **2015**, 7, 34.
17. Zhang, L.; Fu, Y.; Xiong, Z.; Ma, Y.; Wei, Y.; Qu, X.; Zhang, H.; Zhang, J.; Liao, M. Highly Prevalent Multidrug-Resistant Salmonella From Chicken and Pork Meat at Retail Markets in Guangdong, China. *Front. Microbiol.* **2018**, 9, Article 2104.
18. Yang, X.; Wu, Q.; Zhang, J.; Huang, J.; Chen, L.; Wu, S.; Zeng, H.; Wang, J.; Chen, M.; Wu, H.; Gu, Q.; Wei, X. Prevalence, Bacterial Load, and Antimicrobial Resistance of Salmonella Serovars Isolated From Retail Meat and Meat Products in China. *Front. Microbiol.* **2019**, 10, Article 2121.
19. Murmann, L.; dos Santos, M.C.; Cardoso, M. Prevalence, genetic characterization and antimicrobial resistance of Salmonella isolated from fresh pork sausages in Porto Alegre, Brazil. *Food Cont.* **2009**, 20, 191-195.
20. Soltan Dallal, M.M.; Doyle, M.P.; Rezadehbashi, M.; Dabiri, H.; Sanaei, M.; Modarresi, S.; Bakhtiari, R.; Sharifiy, K.; Taremi, M.; Zali, M.R.; Sharifi-Yazdi, M.K. Prevalence and antimicrobial resistance profiles of Salmonella serotypes, Campylobacter and *Yersinia* spp. isolated from retail chicken and beef, Tehran, Iran. *Food Cont.* **2010**, 21, 388-392.
21. Ta, Y.T.; Nguyen, T.T.; To, P.B.; Da Xuan Pham; Le, H.T.H.; Thi, G.N.; Alali, W.Q.; Walls, I.; Doyle, M.P. Quantification, Serovars, and Antibiotic Resistance of Salmonella Isolated from Retail Raw Chicken Meat in Vietnam. *J. Food Prot.* **2014**, 77, 57-66.

22. Sallam, K.I.; Mohammed, M.A.; Hassan, M.A.; Tamura, T. Prevalence, molecular identification and antimicrobial resistance profile of Salmonella serovars isolated from retail beef products in Mansoura, Egypt. *Food Cont.* **2014**, 38, 209-214.
23. Niyomdecha, N.; Mungkornkaew, N.; Samosornsuk, W. Serotypes and antimicrobial resistance of *Salmonella enterica* isolated from pork, chicken meat and lettuce, bangkok and central Thailand. *Southeast Asian J. Trop. Med. Public Health* **2016**, 47, 31-39.
24. Moawad, A.A.; Hotzel, H.; Awad, O.; Tomaso, H.; Neubauer, H.; Hafez, H.M.; El□Adawy, H. Occurrence of Salmonella enterica and *Escherichia coli* in raw chicken and beef meat in northern Egypt and dissemination of their antibiotic resistance markers. *Gut Pathog.* **2017**, 9, 57.
25. Zhu, A.; Zhi, W.; Qiu, Y.; Wei, L.; Tian, J.; Pan, Z.; Kang, X.; Gu, W.; Duan, L. Surveillance study of the prevalence and antimicrobial resistance of Salmonella in pork from open markets in Xuzhou, China. *Food Cont.* **2019**, 98, 474-480.
26. Yoon, R.-H.; Cha, S.-Y.; Wei, B.; Roh, J.-H.; Seo, H.-S.; Oh, J.-Y.; Jang, H.-K. Prevalence of Salmonella Isolates and Antimicrobial Resistance in Poultry Meat from South Korea. *J. Food Prot.* **2014**, 77, 1579-1582.
27. Álvarez-Fernández, E.; Alonso-Calleja, C.; García-Fernández, C.; Capita, R. Prevalence and antimicrobial resistance of Salmonella serotypes isolated from poultry in Spain: Comparison between 1993 and 2006. *Int. J. Food Microbiol.* **2012**, 153, 281-287.
28. Zwe, Y.H.; Tang, V.C.Y.; Aung, K.T.; Alikiiteaga Gutierrez, R.; Ng, L.C.; Yuk, H.-G. Prevalence, sequence types, antibiotic resistance and, *gyr*A mutations of Salmonella isolated from retail fresh chicken meat in Singapore. *Food Cont.* **2018**, 90, 233-240.
29. Cabrera-Diaz, E.; Barbosa-Cardenas, C.M.; Perez-Montaño, J.A.; Gonzalez-Aguilar, D.; Pacheco-Gallardo, C.; Barba, J. Occurrence, Serotype Diversity, and Antimicrobial Resistance of Salmonella in Ground Beef at Retail Stores in Jalisco State, Mexico. *J. Food Prot.* **2013**, 76, 2004-2010.
30. Zdragas, A.; Mazaraki, K.; Vafeas, G.; Giantzi, V.; Papadopoulos, T.; Ekateriniadou, L. Prevalence, seasonal occurrence and antimicrobial resistance of Salmonella in poultry retail products in Greece. *Lett. Appl. Microbiol.* **2012**, 55, 308-313.
31. Yu, T.; Jiang, X.; Zhou, Q.; Wu, J.; Wu, Z. Antimicrobial resistance, class 1 integrons, and horizontal transfer in Salmonella isolated from retail food in Henan, China. *J Infect Dev Ctries* **2014**, 8, 705-711.
32. Hyeon, J.-Y.; Chon, J.-W.; Hwang, I.-G.; Kwak, H.-S.; Kim, M.-S.; Kim, S.-K.; Choi, I.-S.; Song, C.-S.; Park, C.; Seo, K.-H. Prevalence, Antibiotic Resistance, and Molecular Characterization of Salmonella Serovars in Retail Meat Products. *J. Food Prot.* **2011**, 74, 161-166.
33. Thung, T.Y.; Mahyudin, N.A.; Basri, D.F.; Wan Mohamed Radzi, C.W.J.; Nakaguchi, Y.; Nishibuchi, M.; Radu, S. Prevalence and antibiotic resistance of *Salmonella* Enteritidis and *Salmonella* Typhimurium in raw chicken meat at retail markets in Malaysia. *Poult. Sci.* **2016**, 95, 1888-1893.
34. Li, Y.-C.; Pan, Z.-M.; Kang, X.-L.; Geng, S.-Z.; Liu, Z.-Y.; Cai, Y.-Q.; Jiao, X.-A. Prevalence, Characteristics, and Antimicrobial Resistance Patterns of Salmonella in Retail Pork in Jiangsu Province, Eastern China. *J. Food Prot.* **2014**, 77, 236-245.
35. Cai, Y.; Tao, J.; Jiao, Y.; Fei, X.; Zhou, L.; Wang, Y.; Zheng, H.; Pan, Z.; Jiao, X. Phenotypic characteristics and genotypic correlation between Salmonella isolates from a slaughterhouse and retail markets in Yangzhou, China. *Int. J. Food Microbiol.* **2016**, 222, 56-64.
36. Abd-Elghany, S.M.; Sallam, K.I.; Abd-Elkhalek, A.; Tamura, T. Occurrence, genetic characterization and antimicrobial resistance of Salmonella isolated from chicken meat and giblets. *Epidemiol. Infect.* **2015**, 143, 997-1003.
37. Van, T.T.H.; Moutafis, G.; Istivan, T.; Tran, L.T.; Coloe, P.J. Detection of Salmonella spp. in Retail Raw Food Samples from Vietnam and Characterization of Their Antibiotic Resistance. *Appl. Environ. Microbiol.* **2007**, 73, 6885–6890.
38. Nghiem, M.N.; Nguyen, V.T.; Nguyen, T.T.H.; Nguyen, T.D.; Vo, T.T.B. Antimicrobial resistance gene expression associated with multidrug resistant *Salmonella* spp. isolated from retail meat in Hanoi, Vietnam. *Int. Microbiol.* **2017**, 20, 85-93.
39. Bacci, C.; Lanzoni, E.; Vismarra, A.; Alpigiani, I.; Nuvoloni, R.; Bonardi, S.; Brindani, F. Antibiotic resistance and resistance genes in Salmonella enterica isolated from pork meat and pig carcasses in Northern Italy. *Large Anim. Rev.* **2014**, 20, 201-207.
